# Reprogramming of glucose metabolism via PFKFB4 is critical in FGF16-driven invasion of breast cancer cells

**DOI:** 10.1042/BSR20230677

**Published:** 2023-08-02

**Authors:** Swarnali Kar, Nilanjana Maji, Kamalika Sen, Stuti Roy, Atanu Maity, Shubhra Ghosh Dastidar, Somsubhra Nath, Gautam Basu, Moitri Basu

**Affiliations:** 1 Department of Biophysics, Bose Institute, P 1/12, C.I.T. Scheme VIIM, Kolkata 700054, India; 2 Bioinformatics Centre, Bose Institute, P 1/12, C.I.T. Scheme VIIM, Kolkata 700054, India; 3 Basic and Translational Research Division, Saroj Gupta Cancer Centre and Research Institute (SGCC & RI), Kolkata 700063, India

**Keywords:** Aerobic glycolysis, breast cancer, cell invasion, epithelial-to-mesenchymal transition, fibroblast growth factor 16 (FGF16), PFKFB4

## Abstract

Fibroblast growth factors (FGFs) are expressed in both developing and adult tissues and play important roles in embryogenesis, tissue homeostasis, angiogenesis, and neoplastic transformation. Here, we report the elevated expression of FGF16 in human breast tumor and investigate its potential involvement in breast cancer progression. The onset of epithelial–mesenchymal transition (EMT), a prerequisite for cancer metastasis, was observed in human mammary epithelial cell-line MCF10A by FGF16. Further study unveiled that FGF16 alters mRNA expression of a set of extracellular matrix genes to promote cellular invasion. Cancer cells undergoing EMT often show metabolic alteration to sustain their continuous proliferation and energy-intensive migration. Similarly, FGF16 induced a significant metabolic shift toward aerobic glycolysis. At the molecular level, FGF16 enhanced *GLUT3* expression to facilitate glucose transport into cells, which through aerobic glycolysis generates lactate. The bi-functional protein, 6-phosphofructo-2-kinase/fructose-2, 6-bisphosphatase 4 (PFKFB4) was found to be a mediator in FGF16-driven glycolysis and subsequent invasion. Furthermore, PFKFB4 was found to play a critical role in promoting lactate-induced cell invasion since silencing PFKFB4 decreased lactate level and rendered the cells less invasive. These findings support potential clinical intervention of any of the members of FGF16-GLUT3-PFKFB4 axis to control the invasion of breast cancer cells.

## Introduction

The conversion of a benign epithelial tissue toward a fully malignant carcinoma entails extensive remodeling in tissue architecture, driven by both genetic and micro-environmental factors [1]. Epithelial–mesenchymal transition (EMT), an embryonic trans-differentiation program, contributes largely in cancer progression by converting primary tumor cells into invasive in nature [2]. During EMT, notable cellular changes include loss of cell adhesions, change in cell polarity, interactions between new plasma membrane receptors and remodeled extracellular matrix (ECM) components; all finally lead to cell migration and generation of a more plastic mesenchymal cells [2,3]. This change in cellular phenotypes essentially involves the remodeling of the ECM components including collagens, laminin, fibronectin, proteoglycans etc., through a series of qualitative and quantitative changes. This ECM remodelling thus appears to be a key step in facilitating tumor growth and spread by interfering with cell–cell adhesion, polarity, and by augmenting growth factor signaling [4].

In normal cells, multiple metabolic cycles are always operative which provide constant supply of energy and biomass. However, this metabolic program is altered in cancer cells to sustain their continuous growth/proliferation and to adapt to new microenvironment at distant location [5]. A high rate of glycolytic flux, even in the presence of functional mitochondria and oxygen, is a typical metabolic hallmark of tumors. Although this ‘aerobic glycolysis’ is actually less energy-efficient pathway than oxidative phosphorylation, this reprogrammed metabolism confers several growth advantages on cancer cells including cell death evasion, cell proliferation, angiogenesis, and metastasis [6,7]. Thus, the phenomenon of metabolic alterations appears to facilitate successful spread and growth of metastatic tumour at distant sites [5]. The molecular mechanism underlying this metabolic reprogramming of cancer cells is complex and unique for each cancer type. Pinpointing the altered metabolic step can lead to more effectively targeting the metabolism of cancer patient with the consequence of reduced cancer invasiveness.

Gradual acquisition of cell invasiveness can take place due to sequential genetic and epigenetic changes, triggering induction of EMT in response to micro-environmental cues, for example secreted growth factors [8]. The fibroblast growth factor (FGF) family, comprising of 22 ligands, exerts their functions through four, highly conserved, cell–surface tyrosine kinase receptors (FGFR1, FGFR2, FGFR3 or FGFR4) [9]. FGF/FGFR signaling has established roles in the control of multiple biological processes, such as endocrine homeostasis, wound repair, cellular proliferation, differentiation, and survival [10]. However, FGF/FGFR pathway has been demonstrated to have a significant relevance in tumor growth, metastasis, and resistance to anticancer therapies [11–14]. Aberrant FGF/FGFR activation may occur either in ligand-dependent or -independent manner in several cancer types, including breast cancer. Overexpression of FGF ligands cause excessive mitogenic signaling through FGFR, promoting cancer progression. Example includes the elevated expression of FGF2 [15] and its involvement in driving basal-like breast cancer through the autocrine activation of FGFR signaling [16]. FGF2 could also promote BC growth in hormone-independent manner, leading to endocrine therapy resistance [17]. Fillmore et al. has already demonstrated the potential involvement of paracrine FGF9/FGFR signaling in the estrogen-mediated expansion of a breast cancer stem-cell-like subpopulation *in vitro* [18]. FGF16, a member of FGF9 sub-family, is a 207-amino acid protein with a core region of 120 amino acids that acts through binding to heparin and cell surface FGF receptor (FGFR) [19]. Recently, FGF16 is implicated in the progression of hepatocellular [20], lung [21], and embryonic carcinoma [22] with unique mechanistic action for each cancer type. In continuation to these findings, we attempted to investigate the potential oncogenicity of FGF16 and the underlying mechanism in breast cancer progression.

In the present study, the oncogenic potential of FGF16 in breast cancer progression has been demonstrated, where the activation of EMT was found to be critical. Further, it was unraveled that a set of genes involved in ECM remodeling is regulated by FGF16. Detail studies uncovered a hitherto unidentified role of FGF16 in promoting aerobic glycolysis where a robust expression of PFKFB4 was particularly observed. Overall, our findings provide a basis for developing a new metabolism-targeted therapeutic intervention to reduce aggressiveness of breast cancer.

## Results

### FGF16 induces EMT in breast cancer cells

**Figure 1:**
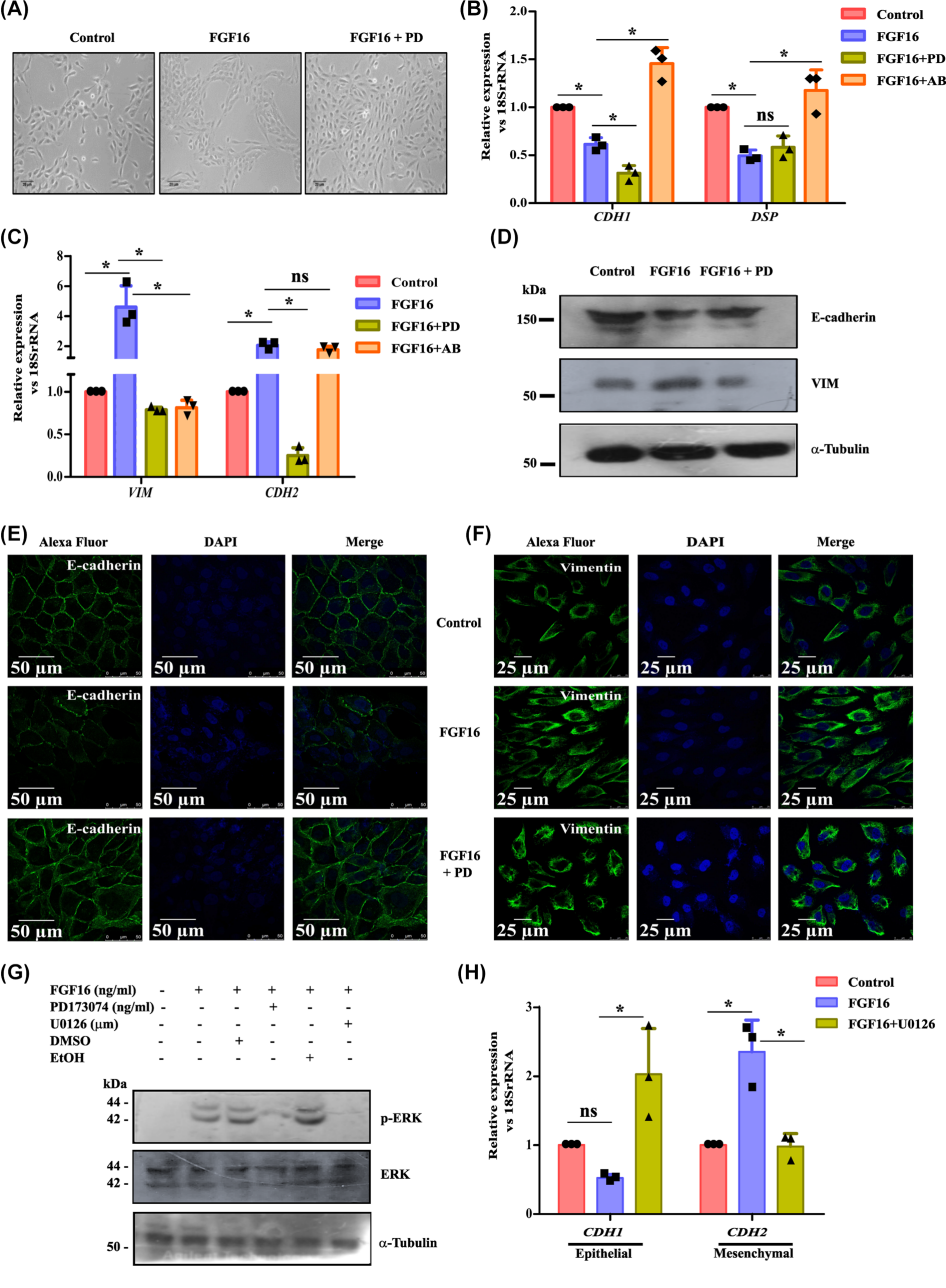
FGF16 induces EMT in breast cancer cells (**A**) MCF10A cell-line was treated with 0.1% BSA in PBS (vehicle) and FGF16 (alone or in combination with PD173074) for 48 h and the morphological changes were captured under microscope. Scale bar: 20 µm. (**B,C**) Quantitative determinations of epithelial (**B**) and mesenchymal (**C**) markers were done by Q-PCR assay with RNA isolated from MCF10A. Relative gene expression is indicated as ‘fold’ change in the *y*-axis (mean ± SD) with statistical analysis by ANOVA followed by Tukey’s Honest Significant Test (HSD). * denotes *P*<0.05, ‘ns' stands for non-significant change. (**D**) Western blot was performed using specific antibodies with lysates of vehicle-, FGF16- and FGFRI-treated MCF10A cells. (**E,F**) Immunostaining with antibodies against E-cadherin and vimentin followed by Alexa Fluor-488 (green) and nuclei staining with DAPI was done in control (vehicle-treated) and FGF16-treated MCF10A cells. Magnification scale bar: 50 µm (E-cad), 25µm (Vim). (**G**) Western blot was performed using specific antibodies with cell lysates after respective treatment. (**H**) The change in gene expression was assessed by Q-PCR assay and relative gene expression is indicated as ’fold’ change in the y-axis (mean ± SD) with statistical analysis by ANOVA followed by Tukey’s Honest Significant Test (HSD) test. * denotes *P*<0.05, ‘ns’ stands for non-significant change.

FGF signaling has been implicated in pathogenesis of many cancers [23]. To investigate the potential role of FGF16 in breast cancer development, we explored whether it can trigger EMT with immortalized human mammary epithelial cell-line MCF10A. In comparison with vehicle-treatment, cells treated with rhFGF16 exhibited morphological alteration, shifting from round cobblestone-like to fibroblast-like elongated shape (Figure 1A). At the molecular level, reduced expression (by ∼50%, *P*<0.05) of epithelial markers like desmoplakin (*DSP*) and E-cadherin (*CDH1*), and significant increase in mesenchymal markers like vimentin (*VIM*) and N-cadherin (*CDH2*) was observed in FGF16-treated cells (Figure 1B,C). Treatment of cells with FGFR-inhibitor, PD173074 (marked as PD), caused a marked reduction in mesenchymal markers and increase in epithelial genes (Figure 1B,C). The same trend was observed in MCF7 cell line (Supplementary Figure S1A). Similarly, combined treatment of FGF16 with its neutralizing antibody reversed the change in expression of EMT markers (Figure 1B,C), supporting a definite contribution of FGF16 in promoting EMT. This reduction in E-cadherin, concomitant with the increase in Vimentin, was reinforced at their protein levels by Western blot (Figure 1D) and confocal imaging (Figure 1E,F and Supplementary Figure S1B). In addition, the reorganization of actin cytoskeleton during morphological change in cells was quite evident from phalloidin staining (Supplementary Figure S1C). Here, pre-treatment of PD along with FGF16 exhibited partial morphological alterations. Further, morphometric analysis of nuclei in DAPI-stained cells revealed that FGF-treated cells had nuclei of significantly larger volume compared with those of control cells (Supplementary Figure 1D). This observation is in agreement with earlier report showing that the alteration in nuclear morphology and architecture are associated with transformation into cancer [24].

**Figure 2:**
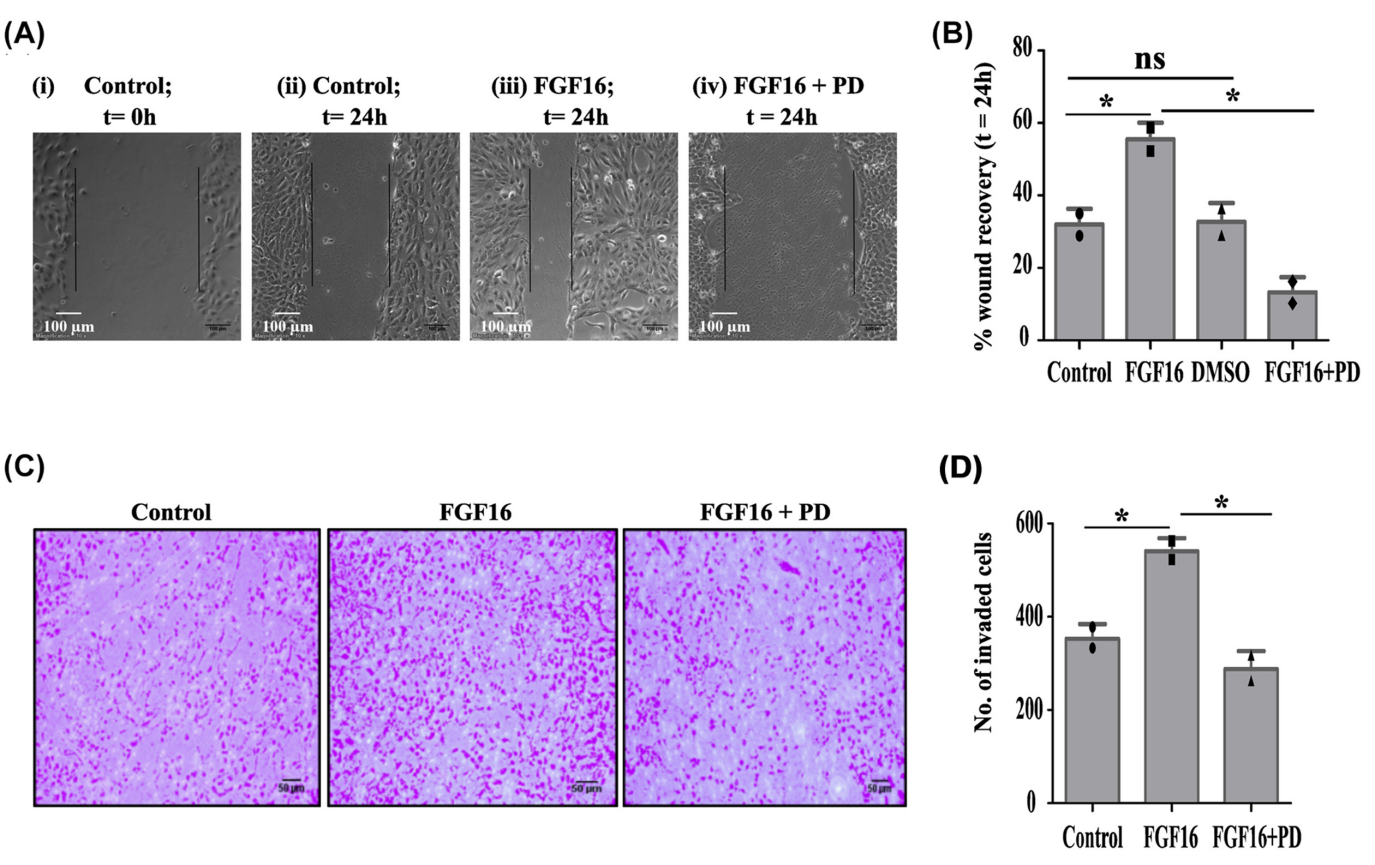
FGF16 promotes cell invasion/migration (**A**) Wound healing assay was performed with MCF10A cells treated with FGF16 alone (iii) or in combination (iv) with PD in the time course of 24 h. *t* = 0 h at control cells in (i) signifies the time of scratching the cells. The boundary of the wound is marked by line which also indicates the width of wound; scale bar: 100 µm. (**B**) The % recovery of the wound for each treated cells compared with control cells at 24 h was calculated and represented with statistical significance calculated by ANOVA followed by Tukey’s Honest Significant Test (HSD) test. * denotes *P*<0.05, ‘ns’ stands for non-significant change. (**C,D**) Transwell invasion assay was performed with MCF10A cells treated as mentioned. Cells at independent fields for each well were counted and plotted with error bar. The statistical significance was calculated by ANOVA followed by Tukey’s HSD test. * denotes *P*<0.05, ‘ns’ stands for non-significant change.

Growth factors, in general, confer their impact on any cellular events, majorly through the activation of signaling pathway(s). To verify this, the activation of P38, MAPK, JNK, and PI3K pathways were assessed and observed the MAPK pathway to be active by 40 ng/ml FGF16 at 1.5 h (Supplementary Figure S1 E,F and Figure 1G). Here, the working dose of PD and U0126 was also determined (Supplementary Figure S1G,H). Subsequent changes in the expression of *CDH1* and *CDH2* genes (Figure 1H) convinced the implication of MAPK pathway in FGF16-induced onset of EMT. Activation of EMT facilitates increased cell motility and invasiveness, key events in cancer metastasis [2,25]. At first, the wound healing assay was performed which exhibited a faster wound closure upon FGF16 treatment compared with vehicle-treated (control) MCF10A cells after 24 h (Figure 2A, compare panels ii and iii), while PD-treatment significantly (*P*<0.05) inhibited FGF16-mediated cell movement (Figure 2A, compare panels iii and iv). The results are quantitatively displayed in Figure 2B. This finding was verified in the MCF7 cell line (Supplementary Figure 1I-J). Similarly, matrigel invasion assay demonstrated that rhFGF16 stimulated invasion of MCF10A cells by ∼1.5 fold (Figure 2C,D, *P*<0.05), while PD-treatment remarkably prevented this invasion.

Since four isoforms make up FGFR, a natural question arises: which of the four isoforms is/are responsible for the early switch in FGF16-activated signaling? This question was addressed by estimating the relative binding affinities of the four isoforms to FGF16 using established *in silico* methods. The starting point was the crystal structure of FGFR1 bound to FGF9 (PDB ID: 5w59). Since FGF9 shares significant sequence homology with FGF16, homology models of FGF16 bound to all four isoforms were built using FGFR1 bound FGF9 as the template. Using the homology models as starting structures, molecular dynamics simulations were performed and the trajectories were analyzed using standard sampling techniques for estimating the relative binding free energies of the four isoforms with FGF16 (see Supplementary Methods, Supplementary Results and Supplementary Table S1) as: ΔΔ*G* = 0.0, 2.2, 3.2 and 7.1 kcal/mol for FGFR1, FGFR2, FGFR3, and FGFR4, respectively. The trend in relative binding affinities was then compared with minor sequence alterations in the four isoforms at the binding interface with FGF16 (Supplementary Figure S2). Binding affinities matched with the sequence alterations, especially FGFR4, with the least predicted affinity showed the maximum variation, suggesting that the relative binding affinities follow the trend: FGFR1 > FGFR2 > FGFR3 >> FGFR4.

**Figure 3:**
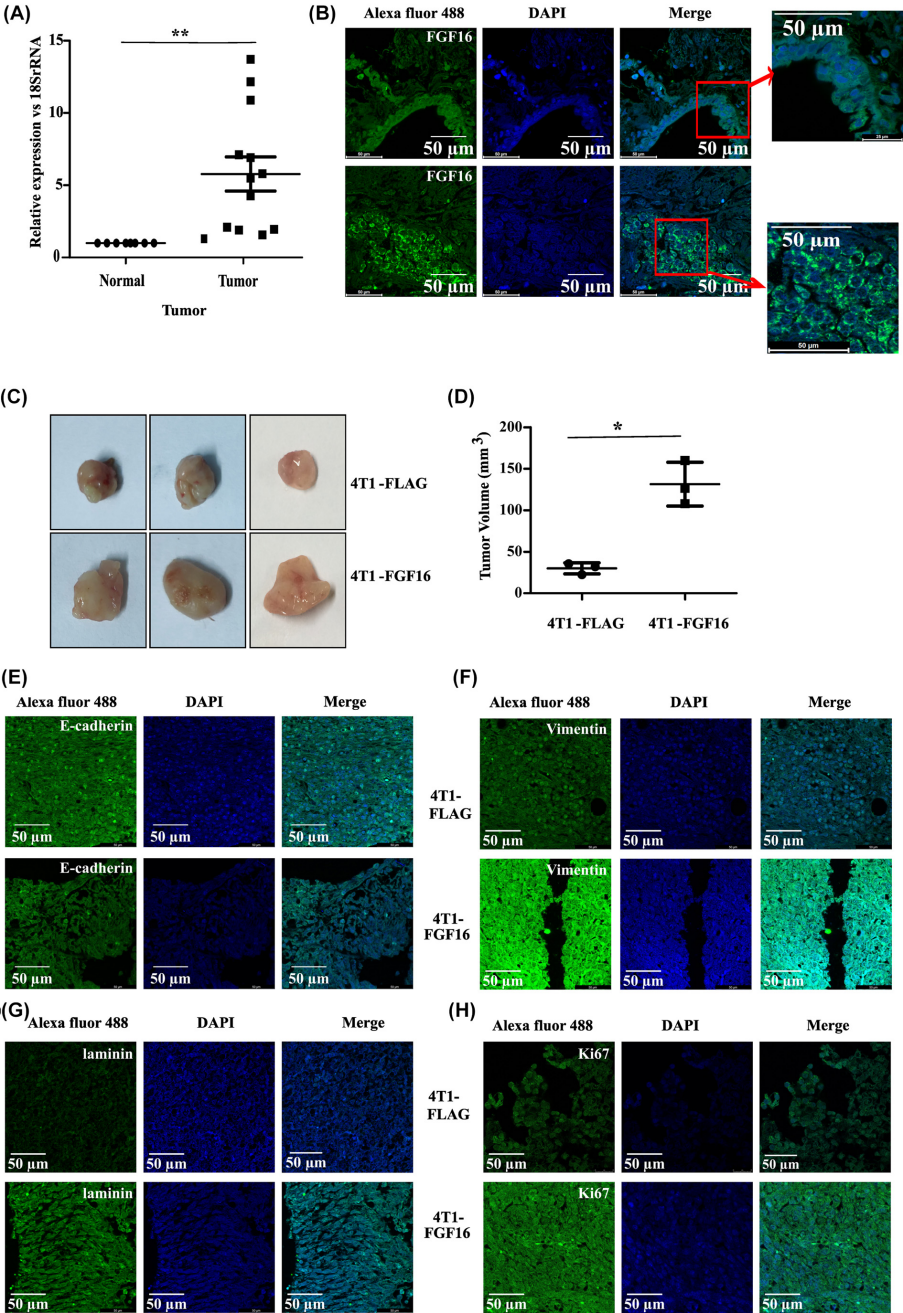
FGF16 supports tumor formation in *in vivo* model (**A**) The expression of *FGF16* at mRNA level was assessed in tissue sections of human breast tumor and adjoining normal region (*n*=13) by Q-PCR assay. Error bar represents mean ± SD with statistical analysis by two-tailed Student’s test. ** denotes *P*<0.005. (**B**) Immunostaining with FGF16-antibody was performed with sections of human normal and cancer tissues. Magnification: 63×; scale bar: 50 μM; zoom factor: 1.5 (for inset). (**C**) 4T1-FLAG (stably transfected with FLAG) and 4T1-FGF16 (stably transfected with FLAG-FGF16) cells were implanted into the mammary fat pad of female BALB/c mice. Primary tumor appeared after four weeks of injection were excised and shown. (**D**) The size of tumor was measured and volume was plotted with statistical significance calculated by two-tailed Student’s *t*-test.* denotes *P*<0.05. (**E-H**) Immunostaining with antibodies against E-cadherin, vimentin, laminin, and Ki67 followed by Alexa Fluor-488 (green) was done with the sections of 4T1-induced tumor. The nuclei stained with DAPI and merged images of Alexa Fluor-488 and DAPI are also shown; scale bar: 50 µm, magnification: 63×.

The clinical relevance of FGF16 expression was investigated. Q-PCR assay (*n* = 5 paired and 8 orphan; Figure 3A; *P*<0.005) and immuno-flourescence-based IHC analysis (*n*=6 Paired; Figure 3B) demonstrated elevated expression of *FGF16* in human breast cancer compared to normal breast tissues. Then, we extended our investigation on xenograft mouse model. Here 4T1-FLAG or 4T1-FGF16 (both stably expressed) was injected subcutaneously into female Balb/c mice and tumors appeared. The size of the tumor, as induced by FGF16, was significantly bigger than that of empty vector transfected set (Figure 3C,D) supporting the contribution of FGF16 in the growth of primary tumor. Immunoflourescence-based IHC analysis demonstrated the reduction in E-cadherin (Figure 3E) and the increase in vimentin (Figure 3F) expression in FGF-induced tumor sections. Furthermore, a remarkable increase in the expression of laminin, a basement membrane protein involved in breast cancer invasion was also evidenced in tumor section (Figure 3G). In addition, prominent staining of Ki67 supported the proliferation of cancer cells in both sets (Figure 3H). Hematoxylin–Eosin staining of the respective tissue sections were performed (Supplementary Figure S3A). In summary, elevated expression in tumor samples and induction of cell invasiveness/migration supported the oncogenic potential of FGF16 in breast cancer.

### FGF16 differentially alters the expression of ECM-associated genes

**Figure 4:**
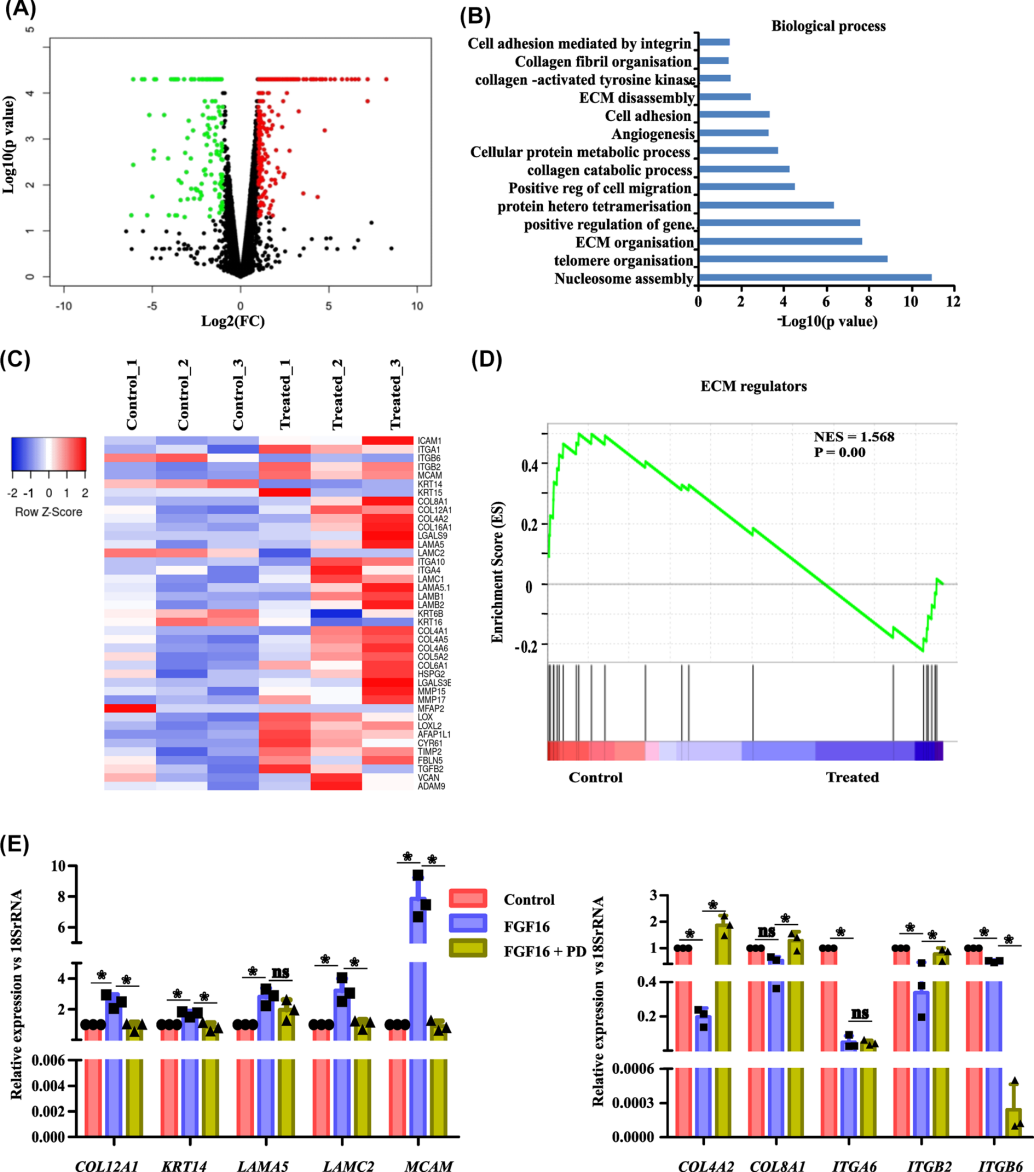
Expressions of ECM-associated genes are altered by FGF16 (**A**) Volcano plot depicts the change in total transcript profile (up-regulated in red, down-regulated in green and non-significant changes in black dots) between control and FGF16-stimulated MCF10A, setting their p-value <0.05 and log2 fold change = 0.58. (**B**) The FGF16-induced up-regulated genes were analyzed by DAVID to identify the affected biological processes. (**C**) Heatmap unveiled differential expression signature of 41 ECM-associated genes from control and FGF16-stimulated sets. The color code of expression values was also given. (**D**) Enrichment plot was generated with differential gene list by GSEA with ‘Canonical Pathways’ gene set from MSigDB. (**E**) Q-PCR assay was performed with RNA isolated from MCF10A, treated with vehicle or FGF16 (alone or in combination with FGFRI). Relative gene expression is indicated as ’fold change’ in the *y*-axis (mean ± SD). The statistical analysis was performed by ANOVA followed by Tukey’s HSD. * denotes *P*<0.05, ‘ns’ stands for non-significant change.

To identify the functional signatures underlying FGF16-induced oncogenicity, whole cell transcript analysis was performed with MCF10A after exposure with FGF16 for 24 h. The treatment of 24 h was selected since the changes in EMT markers were evidenced at this time point (Figure 1). Notably, the expression of a large number of genes was found to be altered whereas several genes remained unchanged at this time point (Figure 4A). Within this list of genes, while applying fold change cutoff of 1.5 (log2 FC >0.58 or < −0.58) and *P*-value <0.05, 956 genes were found to be differentially altered; among them 691 genes were up- and 265 genes were down-regulated (Supplementary Table S2 and 3). Next, DAVID bioinformatic tool was applied and it was found that differentially expressed (DE) genes are involved in a variety of biological processes including angiogenesis, nucleosome assembly, telomere organization, protein metabolic process, ECM disassembly, cell adhesion etc. (Figure 4B). At the same time, pathway analysis by PANTHER Classification System revealed that these DE genes are associated with several cancer-associated signaling pathways including angiogenesis, RAS-pathway(P04393), EGFR signaling pathway (P00018), PDGF (P00047)-, FGF (P00021)-, VEGF(P00056)-, cadherin (P00012)- and integrin-signaling pathway (P00034) and glycolysis (P00024, Supplementary Figure S3B).

Next, we undertook an in-depth analysis of the altered transcription profile. Interestingly, from the total DE gene list, we observed that 41 genes related to extracellular matrix (ECM) organization were deregulated by FGF16 (Figure 4C). Moreover, the enrichment score plot from Gene Set Enrichment Analysis (GSEA) showed a differential distribution of ECM regulator genes (Figure 4D) in FGF16-stimulated cells. Taken together, the bioinformatic analysis of the altered transcription profile revealed a possible role of FGF16 in ECM regulation.

Considering ECM remodeling to be a key determinant of cancer progression, we were motivated to verify the change in a subset of ECM-related genes by Q-PCR assay in the cell-line. Hence, we picked up representative genes of collagen, integrin, laminin, keratin families, all being structural components of ECM. FGF16 stimulation caused up-regulation of *KRT14, MCAM, LAMA5, LAMC2*, and* COL12A1* genes and down-regulation of *ITGB6, ITGB2, ITGA6, COL4A2*, and *COL8A1* genes in MCF10A whereas treatment of receptor inhibitor (RI) with FGF16 somewhat reversed the changes in their expressions (Figure 4E). In continuation, immunofluorescence imaging confirmed the similar change in laminin and Integrin A6 in their protein levels (Supplementary Figure 3C,D) as well. On the contrary, the mRNA level of *ITGA6* and *ITGB6* were induced in the presence of FGF16 in MCF7 cell-line, which could be reverted with PD treatment (Supplementary Figure S3E). Altogether, the regulatory role of FGF16 over ECM related gene expression was established.

### FGF16 induces reprogramming of glucose metabolism

**Figure 5:**
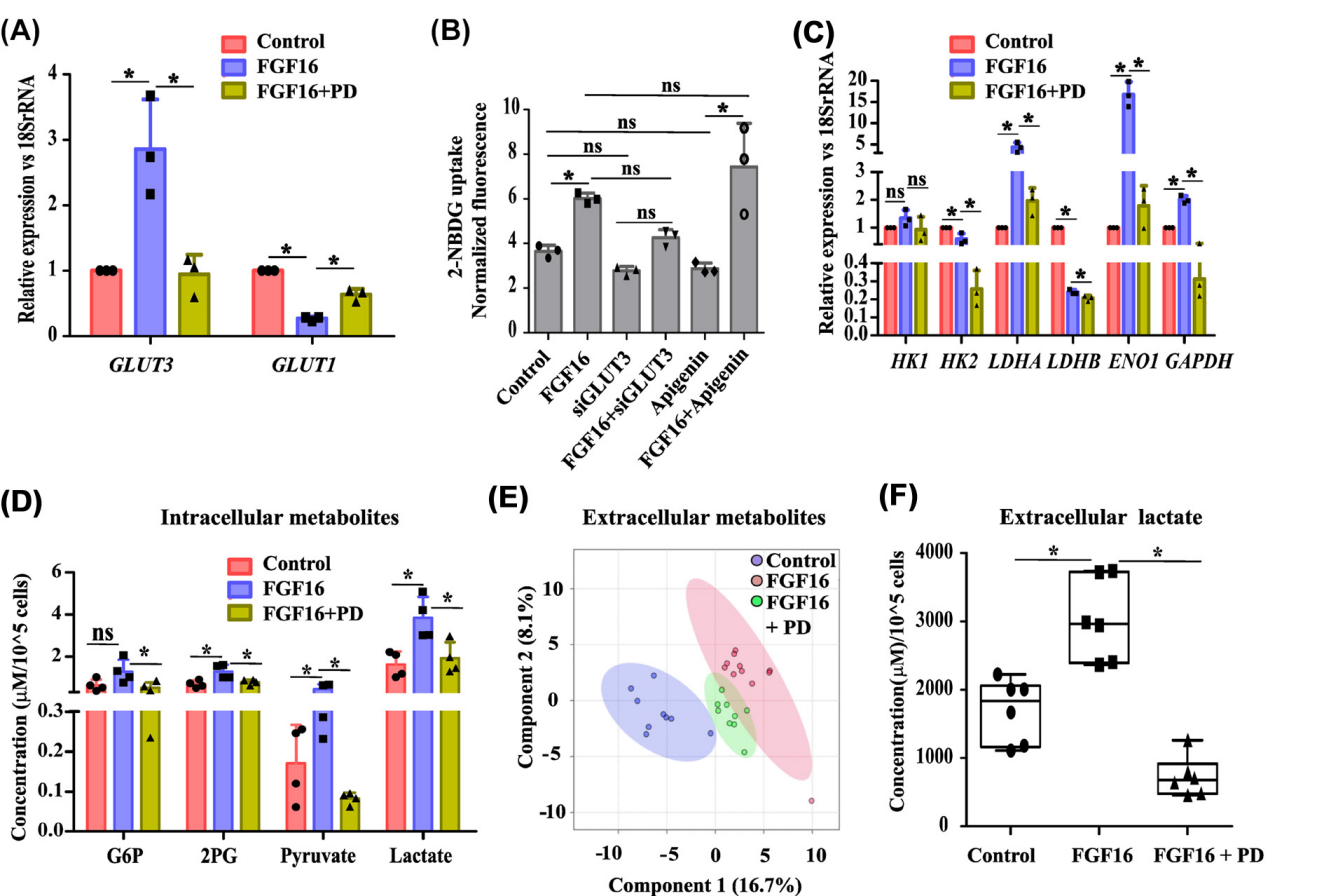
Cellular glucose metabolism is influenced by FGF16 (**A**) Q-PCR assay was performed with RNA isolated from MCF10A cells, treated as mentioned. Relative gene expression is indicated as ‘fold’ change in the *y*-axis (mean ± SD). (**B**) 2-NBDG uptake assay was performed with MCF10A with experimental set up as mentioned. All raw values were normalized to background levels and are presented as the (mean ± SD) of all experiments (*n*=3). (**C**) Change in expression of specific glycolytic genes was quantified by Q-PCR assay with RNA isolated from MCF10A. Relative gene expression is shown as ‘fold’ change in the *y*-axis (mean ± SD). (**D**) Whole cell metabolites were extracted from MCF10A cells after given treatment for 24 h and measured by ^1^H-NMR spectroscopy, quantified with Chenomx NMR Suite 8.3 and plotted with statistical analysis. (**E**) Partial least-squares discriminant analysis (PLS-DA) scores derived from NMR spectra of extracellular metabolites were plotted with Metaboanalyst 3.0 with sample number as follows - control (*n*=9), FGF16 (*n*=11), FGF16 in combination with FGFRI (*n*=9). (**F**) Extracellular lactate was quantified from NMR spectra with Chenomx NMR Suite 8.3 software and plotted with statistical analysis. For A–D and F, statistical analysis was performed by ANOVA followed by Tukey’s HSD. * denotes *P*<0.05, ‘ns’ stands for non-significant change.

Change in the metabolic program is perhaps an emerging driving force to support detachment from ECM to initiate cell migration [5]. Based on our cues on pro-migratory role of FGF16, the change in metabolism was investigated in this regard. Interestingly, RNA-sequencing data revealed a group of transporters i.e. solute carrier (*SLC*) genes to be differentially altered (Supplementary Figure S4A) by FGF16. Among them, a prominent up-regulation in *GLUT3* (*SLC2A3*) was indicated in FGF-treated cells. Q-PCR assay also validated the increase in *GLUT3* by ∼2.5 fold (*P*<0.05) whereas pre-treatment with PD reverted the FGF-induced expression (Figure 5A). In contrast, such alteration was not evidenced in its isoform *GLUT1* by FGF16 (Figure 5A). A similar trend was noticed in the MCF7 cell line as well (Supplementary Figure S4B). Through the up-regulation of *GLUT3* expression, cells enhance glucose uptake and subsequently glycolysis. Hence, 2-NBD-glucose uptake assay was performed in MCF10A. The presence of FGF16 increased the glucose uptake (Figure 5B, *P*<0.05) that was compromised while GLUT3 was down-regulated by siRNA. Moreover, the combination of FGF16 and si*GLUT3* showed the glucose uptake to be lower than FGF16 alone (Figure 5B). In parallel, the uptake was measured in presence of Apigenin, a flavonoid inhibitor of GLUT1 mediated glucose transport [26], to verify the assay performance. Apigenin alone was found to be able to lower the glucose uptake but jointly with FGF16 could retrieve the same extent of glucose uptake as was observed with FGF16 alone (Figure 5B). This finding could indicate the implication of GLUT3 over GLUT1 in FGF16-induced cellular glucose uptake. The knockdown efficiency of siRNA against *GLUT3* was verified by QPCR assay (Supplementary Figure S4C).

Next, the influence of FGF16 on the expression of key glycolytic genes was assessed by Q-PCR assay. A noticeable increase was observed only for *ENO1, GAPDH* by FGF16 that was abrogated by PD pre-treatment (Figure 5C). However, the change in expression of *HK1* and *HK2* was merely remarkable (Figure 5C). Interestingly, between lactate dehydrogenase A and B (*LDHA* and *LDHB*), which govern the production of lactate, *LDHA* was also found to be elevated by ∼5 fold, not observed for *LDHB*, its other isoform (Figure 5C). The same trend was observed in MCF7 as well (Supplementary Figure S4D). Next, the alteration in metabolic profile of MCF10A was examined by 1D proton NMR-spectroscopy. FGF16 enhanced the production of endogenous intermediates of glycolysis (Figure 5D), namely glucose-6-phosphate (G6P), 2-phosphoglycerate (2PG) whereas pre-treatment with PD reversed this induction. Intriguingly, the intracellular level of lactate, the end-product of aerobic glycolysis, was found to be ∼2 fold higher despite a mere increase in pyruvate in FGF16-induced cells (Figure 5D). A prominent reversal in the level of all these intermediates was evident in the presence of PD (Figure 5D). Lactate, after being produced in cells, is mostly secreted in the medium. Hence the metabolic profile of extracellular portion was also examined. Partial least-square discriminant analysis (PLS-DA) of NMR spectral bins of extracellular metabolites demonstrated distinct separation among untreated control cells, cells treated with FGF16 alone or in combination with PD (Figure 5E). Concomitant with the intracellular level, FGF16 increased the secreted lactate concentration by 1.8-fold (*P*<0.05) whereas pre-treatment with PD decreased it by ∼80% (Figure 5F, *P*<0.05). This certainly indicates a FGF16-driven metabolic shift toward aerobic glycolysis concomitant with increase in cell number (Supplementary Figure S4E). Subsequently, we verified whether our earlier observation of induced wound closure by FGF16 is influenced by the increased cell proliferation. After finalizing the working dose of 5FU (Supplementary Figure S4F,G), wound healing assay demonstrated that the treatment of cells with 5-FU could merely affect the wound closure by FGF16 (Supplementary Figure S4H).

### PFKFB4 is critical in FGF16-induced glycolysis and tumorigenesis

**Figure 6:**
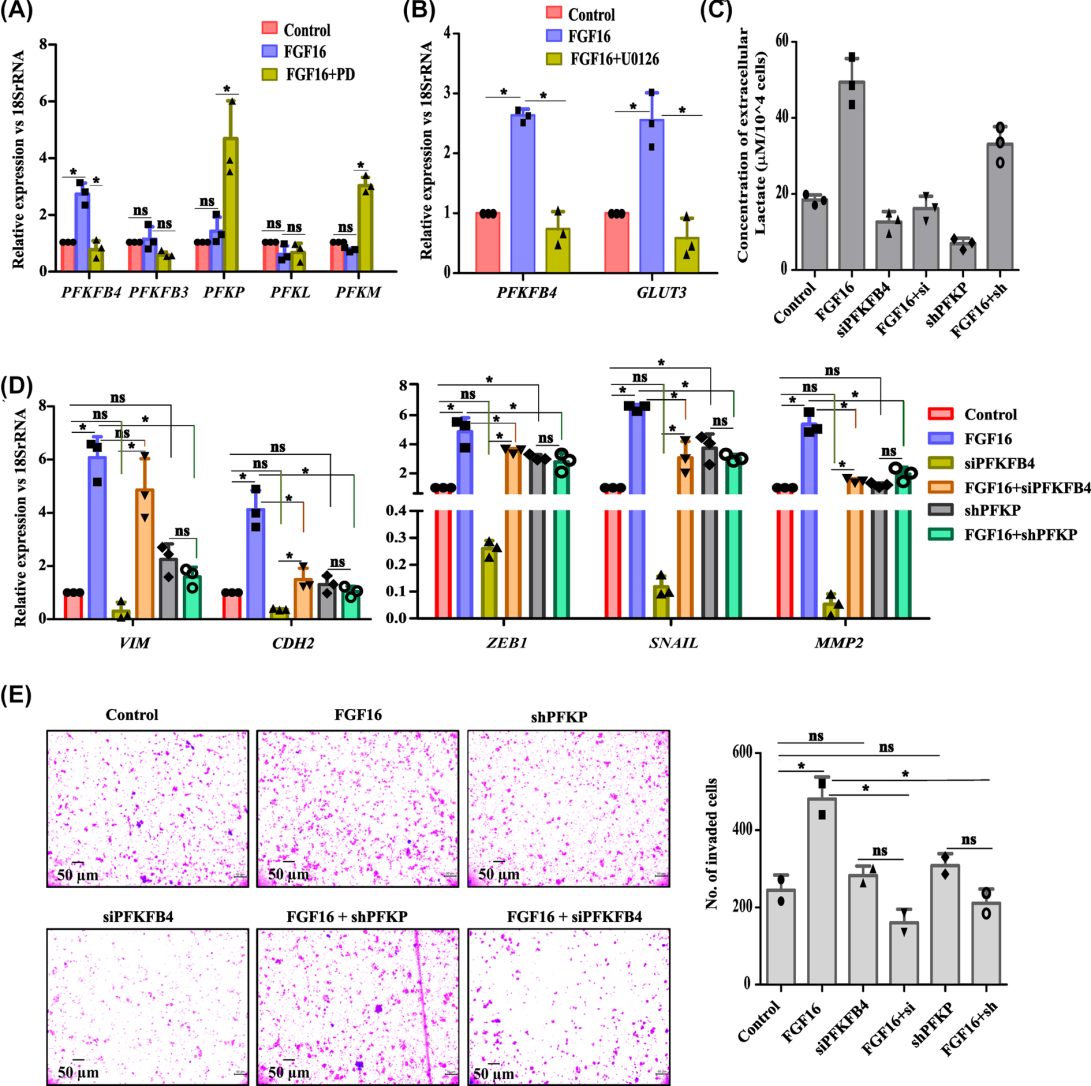
PFKFB4 controls FGF16-induced EMT and cell invasion (**A**) The change in expression of *PFKFB4, PFKFB3, PFKP, PFKL,* and *PFKM* genes was estimated by Q-PCR assay with RNA isolated from MCF10A. Relative gene expression is shown as ‘fold’ change in the *y*-axis (mean ± SD). (**B**) The change in expression of *PFKFB4* and *GLUT3* genes was estimated by Q-PCR assay with RNA isolated from MCF10A. Relative gene expression is shown as ‘fold’ change in the *y*-axis (mean ± SD). (**C**) Extracellular lactate was estimated with experimental set up as mentioned. Lactate concentration was normalized with respect to cell number and plotted with statistical significance. (**D**) Change in expression of genes was quantified by Q-PCR assay with RNA isolated from MDAMB-231 cells. Relative gene expression is shown as ‘fold’ change in the *y*-axis (mean ± SD) with statistical analysis. (**E**) Transwell invasion assay was performed with MDAMB-231 cells treated as mentioned. Cells at independent fields for each well were counted and plotted with error bar and statistical analysis. For A–E, The statistical analysis was performed by ANOVA followed by Tukey’s HSD. * denotes *P*<0.05, ‘ns’ stands for non-significant change.

To establish a mechanistic link between FGF16 and glycolytic induction, the rate-limiting steps of this pathway were investigated. Since our RNA-sequencing data demonstrated a significant up-regulation of *PFKFB4* expression by FGF16, we pinpointed on the members of *PFK* gene family to assess their potential role. Mammalian PFK-1 exists in three isoforms PFKP, PFKL and PFKM. Upon FGF16 over-expression, only *PFKP* showed a modest increase in its mRNA level, while the other two isoforms (*PFKL* and *PFKM*) were slightly decreased (Figure 6A). Two isozymes of PFK2, i.e. PFKFB3 and PFKFB4, showed elevated expression in many cancer types and are involved in altered glycolysis [27]. Interestingly, the expression of *PFKFB4* was significantly enhanced by FGF16 (Figure 6A) whereas PD treatment abrogated the same. Here, the observation of *PFKFB3* to be slightly decreased by FGF16 led us to hypothesize PFKFB4 to be a potential player in the control of FGF16-mediated glycolytic induction (Figure 6A). This finding was verified with MCF7 cell line (Supplementary Figure S5A). Western blot assay demonstrated a similar trend in their protein levels (Supplementary Figure S5B). Detail study uncovered that *GLUT3* and *PFKFB4* genes were turned on by the activated MAPK-signaling pathway as U0126-tretament reversed the FGF16-induced observation (Figure 6B). Subsequently, lactate assay was performed where an increase in extracellular lactate content was observed by FGF16 (Figure 6C), typical to our earlier finding (Figure 5F). Surprisingly, siRNA-mediated abrogation of *PFKFB4* reduced this secretion by FGF16 by >2-fold (Figure 6C, *P*<0.05). However, down-regulation of *PFKP* reduced lactate production *albeit* maintained a moderately higher level in combination with FGF16 (Figure 6C, *P*<0.05).

Furthermore, we deciphered the influence of the FGF16-FGFR-PFKFB4 axis on facilitating the onset of ECM remodelling as well as EMT. For this, the changes in expression of two EMT regulators, *ZEB1* and *SNAIL*, two mesenchymal markers, Vimentin (*VIM*) and N-Cadherin (*CDH2*), and one ECM degrading enzyme, Matrix Metalloproteinase-2 (*MMP-2*) were quantified at their mRNA level in both MDA-MB-231 (Figure 6D) and MCF10A (Supplementary Figure S5C). Noticeable up-regulation of all the five genes was observed by FGF16 (Figure 6D) that was compromised by *PFKFB4*-siRNA. In contrast, suppression of PFKP resulted in the up-regulation of all the five genes and maintained the very same trend in combination with FGF16 (Figure 6D) as observed with FGF16 alone. Similar trend in the change in expression of these selected genes was found in MCF10A (Supplementary Figure S5C) as well. This signifies that FGF16 and PFKFB4 share an equivalent mode of action to augment cell motility at molecular level. The knockdown efficiencies of shRNA and siRNA, against *PFKP* and *PFKFB4*, respectively, were verified by QPCR assay and Western Blot (Supplementary Figure S5D,E). This investigation was extended at the phenotypic level by matrigel invasion assay in MDA-MB-231 because of its aggressive nature. Here, FGF16-driven cell invasiveness was reduced by ∼50% (*P*<0.05) when *PFKFB4* expression was abrogated by siRNA (Figure 6E). In contrast, sh*PFKP* could not notably affect the cellular invasion alone or in combination with FGF16. Overall, our findings support the critical involvement of PFKFB4 isoform in FGF16-promoted metabolic shift followed by cell motility.

## Discussion

The major cause of fatality in breast cancer is its aggressiveness, achieved through increased motility/invasion of cells, promoted by growth factors and cytokines, secreted from tumor micro-environmental cells. Concurrently, reactivation of EMT in adult cells triggers localized invasion and hence is considered to be the first step in metastatic dissemination [28] and is correlated, to some extent, to poor patient survival [29–31]. This transient up-regulation of EMT-inducing transcription factors occurs as a response to the activation of intracellular signaling pathways by various growth factors including FGF [32]. Therefore, FGF signaling has been implicated in promoting the oncogenic progression [10]. Apart from developmental role in cardiomyocytes [33], FGF16 has been identified to be potentially involved in hepatocellular carcinoma [20] as well as in lung cancer [21]. Our investigation was initiated with the finding of elevated expression of FGF16 in human breast tumor samples. Detail study uncovered its involvement in the activation of MAPK signaling cascade leading to onset of EMT followed by cell invasion (Figures 1 and 2). In conjunction, key components of ECM were found to be altered (Figure 4) which may, in turn, support tumor growth and spread. Moreover, gradual growth in tumor size reinforced the oncogenic potential of FGF16. High expression of FGF8 has been implicated in promoting EMT and tumorigenesis in oral squamous carcinoma cells and in colorectal cancer [34,35]. Our finding further supports the emerging evidences demonstrating atypical expression of FGFs and aberrant FGF/FGFR signaling in cancer development.

Whole cell transcriptomic analysis unravelled that a group of genes of solute carrier (*SLC*) family is differentially regulated by FGF16, indicating a possibility of metabolic alteration, an oncogenic hallmark. The SLC superfamily encodes membrane transport proteins for exchanging molecules including glucose, amino acids, vitamins, nucleotides, inorganic ions, organic ions, neurotransmitters and drugs [36]. Altered expression of *SLC* genes has been demonstrated to affect various steps of tumorigenesis, including proliferation, apoptosis, invasion and metastasis, chemotherapy resistance [37]. Even SLCs have been considered to be a therapeutic target in the control of pancreatic and renal cell carcinoma [38,39]. Surprisingly, the elevation of mRNA level of *GLUT3* (*SLC2A3*), a transporter with highest affinity for glucose, but not of its isoform *GLUT1*, was observed (Figure 5) and eventually the increase in glucose uptake by FGF16. High expression of *GLUT3* has been demonstrated in brain cancer [40] and is responsible to resist certain chemotherapy drugs as well [41]. The essential role of GLUT3 in metabolic alteration to promote breast cancer brain metastasis is also established [42]. In addition, it is known that elevation of GLUT3 promotes EMT, invasion, metastasis and inflammation in triple negative breast cancer [43].

Concomitant with the elevation of glucose uptake, the glycolytic flux was found to be increased with the secretion of lactate (Figure 5). Whole cell metabolomics and expression profiling of glycolytic genes revealed that cells preferred glycolysis over TCA cycle under the influence of FGF16 (Figure 5). The intermediates of the glycolytic pathway are utilized in multiple biosynthetic processes and NADPH in sequestering reactive oxygen species to resist cell death [44]. Aerobic glycolysis is known to be one of the most prominent metabolic changes associated with cellular transformation into cancer. Lactate is not only a mere waste product of aerobic glycolysis; it is actually exploited through the onset of oncogenesis and cancer progression. Higher lactate production has been documented in pancreatic ductal adenocarcinoma [45], renal cell carcinoma [46], breast cancer [47], and lung cancer cells [48]. Lactate promotes invasion by tumor acidification, continuous proliferation by activating ‘metabolic symbiosis’, evasion of immune system and angiogenesis [49]. Further, lactate specifically assists EMT by reducing the pH of tumor microenvironment. It not only activates MMPs but also stimulates the expression of relevant transcription factors including VEGF, HIF-α, leading to cell migration [50].

In this report, PFKFB4 was unveiled to be specifically up-regulated by FGF16 (Figure 6). Between two PFK families, PFK-1 catalyzes the second rate-limiting step of glycolysis, converting fructose-6-phosphate into fructose-1, 6-bis phosphate. Three isoforms of mammalian PFK-1 exist. While PFKL and PFKM are enriched in liver and muscle, respectively, the third isoform, PFKP, is mainly expressed in platelet and also elevated in most human cancer cells [51,52]. PFKFB4 is also an emerging contributor in many cancer types including lung adenocarcinoma [53], breast cancer [54–57] and gastric cancer[58]. Apart from glycolysis, PFKFB4 governs cancer progression through various mechanisms-regulating transcription through phosphorylating SRCs [53], affecting autophagy [27] or inducing the production of hyaluran, a major constituent of ECM [56]. Mechanistically, PFK-1 is allosterically inhibited by several products of glucose metabolism including ATP, citrate, and H^+^ ions and is activated by fructose-2,6-bisphosphate (F26BP), a shunt product of glycolysis. F26BP has the ability to counteract the allosteric inhibition by ATP, thus stimulating PFK1, glucose uptake and flux through the entire glycolytic pathway as well [59]. The intracellular concentration of F26BP is controlled by a family of four bi-functional enzymes (PFKFB1–4), having both fructose-6-phosphate kinase and F26BP phosphatase activities [60]. The increased production of lactate by FGF16, a key highlight of our investigation, was mediated by PFKFB4 (Figure 6). Even FGF16-induced EMT/invasion of cells was found to be lowered while abrogating the expression of *PFKFB4* (Figure 6). On contrary, these molecular changes remained unperturbed by *PFKP* knockdown (Figure 6). However, direct regulation between PFKFB4 and transcriptional machinery of EMT genes, if any, remained less ventured, thus providing a productive field for further investigation.

**Figure 7:**
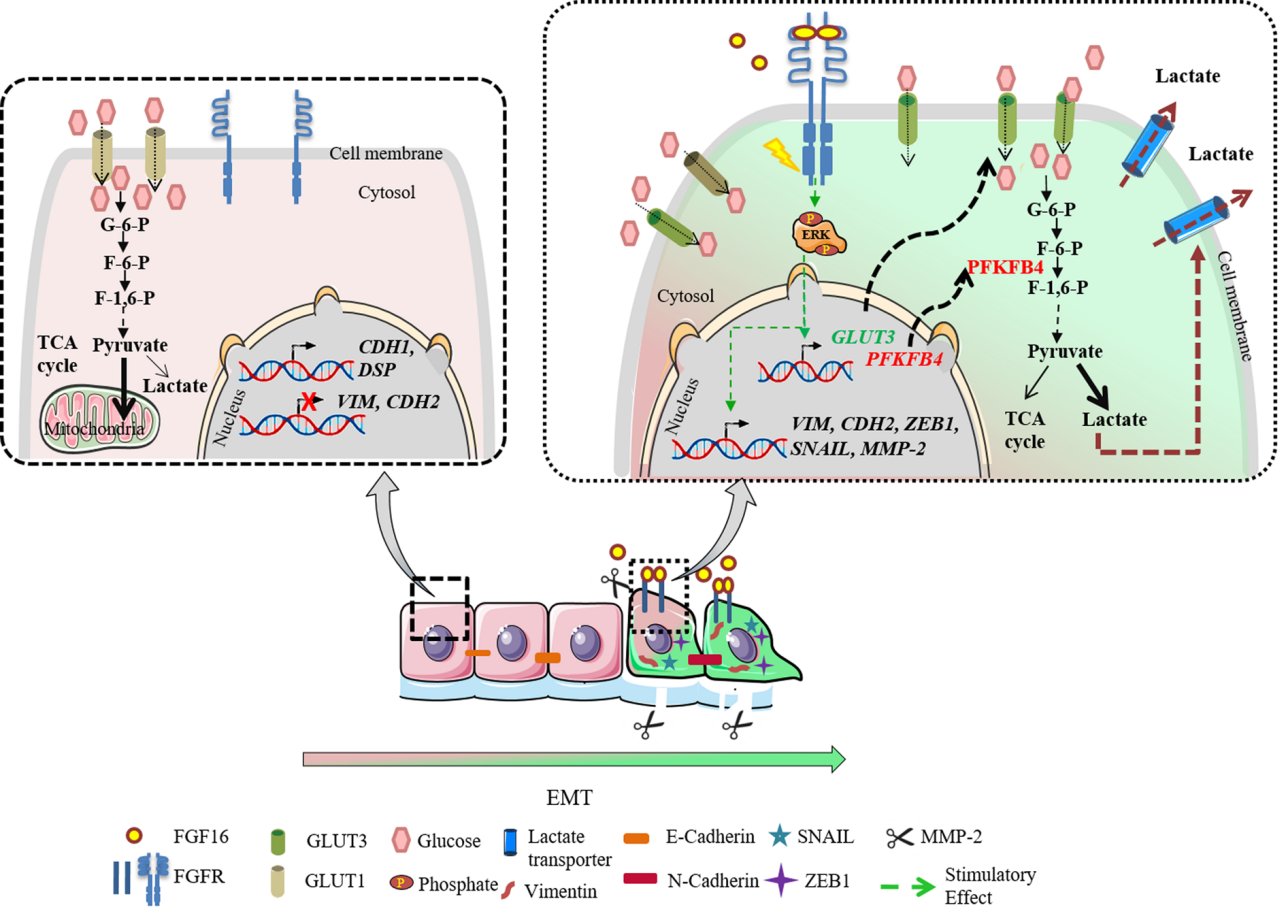
FGF16 rewires the whole cell transcription and metabolism to instigate EMT The impact of activation of FGF16-GLUT3-PFKFB4 axis was shown schematically. In absence of FGF16, expression of E-cadherin, Desmoplakin preserved the ‘epithelial’ status while glucose was mainly metabolized via glycolysis followed by TCA cycle. Activation of FGF16-mediated signaling (MAPK signaling pathway) induced mRNA expression of mesenchymal markers -*VIM, CDH2*, and *SNAIL*. In addition, FGF16 up-regulated the transcription of *GLUT3* and *PFKFB4*. GLUT3 tunneled glucose molecules into cells and the glucose metabolism was inclined more towards lactate production than TCA cycle, through PFKFB4 and mediated the initiation of EMT. (The Figure was partly generated using Servier Medical Art, provided by Servier, licensed under a Creative Commons Attribution 3.0 unported license).

In conclusion, here we present a comprehensive mechanistic investigation on oncogenic potential of FGF16 in breast cancer through the initiation of EMT and invasion. The breast cancer cells were found to adopt the GLUT3–PFKFB4 axis under influence of FGF16 (Figure 7). Up-regulation of *GLUT3* and *PFKFB4* results in increased glucose fuelling in the cells coupled with elevated lactate production and secretion, indicating a metabolic shift towards glycolysis (Figure 7). This finding has the potential to open up a new avenue to design novel therapeutic agent(s) against FGF16–GLUT3–PFKFB4 axis that may reduce the invasiveness of breast cancer cells.

## Materials and methods

### Cell culture and treatment

MCF10A was cultured in DMEM/F12 (Gibco) supplemented with 5% horse serum (Gibco), 20 ng/ml EGF (Peprotech, U.S.A.), 0.5 μg/ml hydrocortisone (Sigma-Aldrich, St. Louis, MO), 100 ng/ml cholera toxin (Sigma), 10 μg/ml insulin (Sigma), and 1% penicillin/streptomycin (Gibco).

MDA-MB-231 and 4T1 cell-lines were obtained from national cell repository at National Centre for Cell Sciences (NCCS), Department of Biotechnology, Government of India, Pune, India. The cells were maintained in Dulbecco’s Modified Eagle’s Medium (DMEM; Gibco, Invitrogen, U.S.A.). MCF7 cells were cultured in RPMI-1640 (Gibco) medium. Both media were supplemented with 10% fetal bovine serum (FBS) (Gibco) and 1% penicillin/streptomycin at 37°C in 5% (v/v) CO_2_.

Cells were starved for 16 h prior to the treatment with recombinant human FGF16 (rhFGF16; Peprotech) at 40 ng/ml for 24 h, unless otherwise mentioned. Treatment with PD173074 (PD; Cayman, U.S.A.), an inhibitor of FGF-receptor, was done at 20 ng/ml for 30 min prior to FGF16 treatment. 0.1% BSA in PBS was used as vehicle for growth factor treatment and DMSO was used as vehicle in ‘control’ cells for PD-treatment. The treatment with the inhibitors was as follows: 5-Fluorouracil (5-FU), a potent cell proliferation blocker, at 50 μM; 2-deoxy glucose, glycolytic inhibitor at 50 mM [61] and U0126, a highly selective inhibitor of MAPK/ERK kinase at 10 μM [62,63]. The control set of each inhibitor was given equal volume of vehicle.

### Cell imaging

MCF10A cells were starved for 16 h, treated with rhFGF16 for 48 h and the change in morphology was visualized and captured by Olympus 1X 83 inverted microscope.

### Expression constructs and transfection

For transient/stable transfection, cell-lines were seeded in 1 × 10^5^ cells/well in 6-well culture plate. After 24 h, 1 µg of construct was transfected using 2 µl Fugene-HD (Promega, Madison, U.S.A.) in Opti-MEM medium (Gibco) and kept for 5 h followed by replacement with fresh complete medium. The cells were incubated for 24 h before performing assays. For stable transfection, cells were selected in G418 (400 µg/ml). The shRNA mediated knockdown of *PFKP* expression was performed by transfection as described above.

### siRNA transfection

The RNA interference was carried out by the siRNAs against *PFKFB4* and non-targeting siRNA at 20 nM and *GLUT3* at 10 nM using 10 µl INTERFERin reagent (Polyplus, USA) on cells seeded in 6-well culture plate. The cells were further incubated for 24 h before performing assays.

### Quantitative real-time PCR (Q-PCR)

Total RNA was isolated from cells using RiboZol^™^ RNA extraction reagent (Amresco) following the standard protocol. First-strand cDNA synthesis was done with RevertAid first strand cDNA synthesis kit (Thermo Fisher Scientific, U.S.A.) followed by Q-PCR assay by using Power SYBR green master mix (Invitrogen). The comparative *C*_T_ method (ΔΔ*C*_T_) was used to measure relative gene expression where the fold enrichment was calculated as: 1/2^ΔΔ*C*^_T_. Here, Δ*C*_T_ is the *C*_T_ of the housekeeping gene subtracted from the *C*_T_ of target gene. ΔΔ*C*_T_ is the Δ*C*_T_ of control sample subtracted from Δ*C*_T_ of treated sample. The fold change >1 means increased expression and <1 means decreased expression. The primer sequences are mentioned in Supplementary Table S4.

### Western blot

Following proper treatment/transfection, whole cell extracts were prepared from cell pellets using RIPA buffer (50 mM Tris-HCl pH 8, 150 mM NaCl, 1% NP40 and 1 mM EDTA) by incubating in ice for 20 min with Protease Inhibitor Cocktail (Roche) followed by sonication. Then, lysed cells were centrifuged at 10,000 rpm for 10 min and supernatants were taken for measuring concentration. The samples were electrophoresed on 7% or 10% or 12% SDS-PAGE and transferred on nitrocellulose membrane (Merck Millipore) followed by blocking with non-fat skimmed milk (3%) for 1 h and probed with specific primary antibodies for overnight at 4°C. For phospho-antibody, blocking was performed in BSA (5%). After incubating with HRP-tagged secondary antibody, blots were developed with Western ECL substrate (Bio Rad, Hercules, CA, U.S.A.). Primary antibodies used are enlisted in Supplementary Table S5.

### Immunocytochemistry

Cells were seeded (8 × 10^4^ cells/well) on coverslips in 6-well plate and grown to 70% confluency. After required treatment/transfection, the cells were fixed with 4% paraformaldehyde for 15 min, permeabilized with 1% Triton X-100 for 10 min and blocked with 3% BSA for 1 h. Cells were then incubated with anti-VIM, anti-E-cadherin, anti-laminin and anti-ITGA6 antibodies for 1.5 h. Following washes with 1× PBS-T buffer for 5 min for three times, the cells were incubated with Alexafluor 488-conjugated secondary antibody for 1 h at RT. For Phalloidin staining, cells were incubated with Alexafluor 488-conjugated phalloidin for 1.5 h. The coverslips were then washed with PBS-T and mounted with Prolong Diamond Anti-fade Mountant with DAPI (P36966, Thermo Fisher Scientific). The images were captured with Leica TCA SP8 confocal microscope with Leica Application Suite (LAS). All the imaging experiments were done in duplicate.

### Nuclear morphometric analysis (NMA)

Nuclei of DAPI-stained (*n*=30) cells, captured by confocal microscope from two independent experiments, were selected for each condition of experiment. Nuclei were marked to assess morphometric data i.e. area, aspect, area box, radius ratio and roundness. ImageJ (NHI, Bethesda, MD, U.S.A., http://rsbweb.nih.gov/ij/) with NMA plug-in (http://www.ufrgs.br/labsinal/nma/) was used. Nuclear Irregularity Index (NII) was calculated for each condition as described in [64] and fold change values with respect to control set was plotted with Graphpad Prism 5.01.

### RNA sequencing and analysis of differentially expressed genes

For RNA-seq analysis, total RNA was extracted from MCF10A cell line using RNAiso Plus (Takara Bio) following standard protocol. The sample was treated with DNase I and its quality was verified by Agilent Bioanalyzer® RNA 6000 Nano/Pico Chip. Samples with an RNA integrity number (RIN) > 7 were further processed. One microgram of total RNA was used for library preparation using a NEBNext Ultra II DNA Library Prep Kit for Illumina (New England Biolabs). Sequencing was performed on NovoSeq 6000 at Sandor Speciality Diagnostics Pvt. Ltd. Raw Data Processing was performed by In-house perl scripts which removed low quality bases and adapter sequences. Cufflinks (Module: Cuffdiff) was used for differential expression analysis with parameters Log_2_FC = 0.58 and *P*-value cut-off <= 0.05. Three biological replicates were used for each sample.

The biological and functional classification of differentially expressed genes was executed by using DAVID biological resources and PANTHER Classification System (http://pantherdb.org/). To get the idea of enrichment of gene sets in a specific biological process in an unbiased manner, Gene set enrichment analysis (GSEA) was performed with GSEA tool (Broad Institute) using default setting.

### NMR spectroscopy

For extracellular metabolomics, the media from transfected/treated cells were mixed with ice-cold methanol: chloroform (1:1) solution, vortexed for 30 s and then subjected to cold centrifugation at 12000 rpm for 20 min at 4°C. Subsequently, the upper aqueous layer was collected, lyophilized and stored at −80°C till further analysis. For intracellular metabolic profiling, cells were washed with ice cold PBS and then immediately quenched with 50% chilled methanol, counted under microscope using haemocytometer and harvested. Cells were then lysed by sonication at amplitude 50 for 1 cycle of 30 sec pulse followed by 20 sec gap followed by addition of chilled chloroform. Then, the sample was vortexed for 5 min and the whole suspension was then centrifuged at 12000 rpm for 20 min at 4°C. The separated aqueous layer was collected and lyophilized properly. Dried samples were then stored at -80°C till use.

NMR spectra were acquired using a Bruker Avance 700 spectrometer operating at 700 MHz and at 298 K. Regular one-dimensional proton NMR spectra were obtained using the pulse program 1D NOESYPR1D with a relaxation delay of 2 s. Spectra were acquired with 512 scans and the obtained FID were processed using a line-broadening factor of 1 Hz and Fourier-transformed. Spectra were manually phased, baseline-corrected, and chemical-shift-referenced to TSP (*δ* = 0.00).

### Analysis of NMR spectra

1D NMR spectra were subjected to multivariate data analysis using the program Metaboanalyst 5.0. The data were normalized and scaled using Autoscaling. Principal Component Analysis (PCA) was utilized for data overview and outlier detection. The data was modeled with the supervised method of Partial Least Squares - Discriminant Analysis (PLS-DA) using the same scaling and centering parameters.

The metabolites were identified and quantified using an untargeted profiling approach using Chenomx NMR Suite 8.3 Software (Chenomx, Inc., Edmonton, Alberta Canada). Six spectra from each experimental group were considered for quantification of extracellular metabolites and four spectra were used for intracellular metabolite quantification. The concentrations obtained from the spectra were plotted with GraphPad Prism (Version 5.01).

### Glucose uptake assay

Glucose uptake was measured using a cell-based assay kit (#600470, Glucose uptake cell-based assay kit (Cayman Chemical, Ann Arbor, MI)). Briefly, cells were seeded (10000 cells/well) in a black, flat-bottomed 96-well microplate in triplicate. After 24 h, cells were treated with FGF16 alone or in combination with si*GLUT3* or Apigenin, an inhibitor of glucose transport by GLUT1 (1:500) in glucose-free medium. Following 24-h incubation, cells were incubated with 100 μg/ml 2-NBDG (a fluorescently-labeled deoxy-glucose analog) for 30 min at 37°C in a humidified atmosphere of 5% CO_2._ The cells were washed with PBS and the fluorescent signal was measured using Varioskan Flash Spectral Scanning Multimode Reader (Thermo Scientific). The experiment was done in three independent sets.

### Lactate assay

Lactate excreted by cell-line upon respective treatment/transfection in culture media was measured using a plate-based colorimetric assay kit (MET-5012, Cell Biolabs). Briefly, cells seeded in 96-well microplate were transfected with FGF16 alone or in combination with si*PFKFB4* or sh*PFKP*. Post-incubation of 24 h, exhausted media was collected and processed according to the manufacturer’s protocol. Lactate concentration was normalized with respect to cell number.

### Trypan Blue exclusion assay

A total of 1 × 10^5^ cells/well were seeded in a 12-well plate. After 24 h, cells were treated with FGF16 alone or in combination with 2-DG or 5-FU. Next day, cells were counted before and after Trypan blue staining using haemocytometer. The experiment was performed in triplicate. Cell viability was calculated using the following formula: % viable cells = [1.00 − (Number of blue cells ÷ Number of total cells)] × 100

### Syngeneic mouse model

Female Balb/c mice (6-8 weeks old) were injected into mammary fat-pad with 1 × 10^6^ 4T1 cells (stably transfected with FLAG/FLAG-FGF16 constructs) suspended in 0.1 ml of PBS. After 10 days, primary tumors were visible and the tumor volume was monitored and measured (twice a week) for next 20 days followed by Avertin mediated anesthesia of mice and their sacrifice by cervical dislocation. The tumors were excised, measured and collected in formalin prior to prepare paraffin blocks. All animals were treated in accordance with the guidelines of the Committee on the Care and Use of Laboratory Animals of the Institute of Animal Resources and Institutional Animal Care and Use Committee (IACUC) and the ethical approval was obtained from central animal house and research facility, Bose Institute. For each group, 5 mice were used; among which tumor of three representative images were given. Entire animal experiment was done at animal house, Bose Institute. Tumor volume was calculated by the modified ellipsoidal formula: *V* = ½ (Length × Width^2^).

### Immunohistochemistry (IHC)

Immunohistochemical analysis was done using 5 µm thick paraffin-embedded sections of tumor xenograft. At first, the slides were de-waxed by heating at 70°C for 30 min followed by 5 min wash in xylene and rehydration with 100%, 90%, 70%, 50% ethanol up to pure distilled water. For heat-induced antigen retrieval, the sections were then kept in 10 mM citrate buffer (65°C) followed by wash with PBS and blocking in 3% FBS in 1× PBS for 1 h. Staining was done with anti-Laminin (1:400), anti-Ki67 (1:500), anti-E-cadherin (1:400) and anti-Vimentin(1:100) antibodies diluted in 1XPBS containing 1% FBS for 2h. After proper washing, the slides were incubated with the secondary antibody (Alexa Fluor-488 conjugated; 1:500) for 1.5 h followed by washing with PBS-T and staining with DAPI. The images were captured using Leica TCS SP8 microscope and LAS X software.Haematoxylin-Eosin staining of the sections was performed by following the standard protocol by professional pathologists.

### Wound healing assay

Cells at 70% confluency were treated/transfected as mentioned above and prior to that, scratching was carried out with a 10 μl pipette tip (mentioned as *t* = 0 h at the figures). Cells were washed several times with PBS to remove the detached ones and supplied with new growth medium. Photographs of the scratches were taken at 0 h and 24 h with 10× objective lens of inverted microscope (Olympus 1X 83) equipped with in-built camera and software (CellSens). The images of 5FU-treated set were captured using 20× objective of the microscope equipped with camera LEICA DFC450C and in-built software (Leica Application Suite).The length of wound for all the sets was measured by ImageJ software. The experiment was performed in duplicate.

### *In vitro* invasion assay

Corning® BioCoat™ Matrigel® Invasion Chamber (Catalog No. 354481) was used to assess *in vitro* invasion by FGF16. Briefly, 1 × 10^5^ cells were added in serum-free medium in the upper chamber and allowed to invade in presence of either 10% FBS or rhFGF16 alone or in combination with PD173074 in the lower chamber.

To check the effect of PFKFB4 and PFKP, cells were first transfected with respective siRNA/shRNA and next day were trypsinized, counted and seeded in upper chamber to allow the cells to invade in presence of 10% FBS. After incubating for 22 h at 37°C in 5% CO_2_, the invaded cells on the lower surface were fixed in methanol, stained with crystal violet and counted under microscope (Leica Microsystems). The images were captured with 20× objective of the microscope equipped with camera LEICA DFC450C and in-built software (Leica Application Suite). The experiment was performed in duplicate.

### Clinical analysis with human patient tissue samples

Primary malignant breast tumor and adjacent normal tissue specimens were collected from affected individuals with informed written consent. The study was approved by the Institutional Ethics Committee of Saroj Gupta Cancer Centre and Research Institute, under regulation of the Govt. of India (Registration No. ECR/250/Inst/WB/2013/RR-20). All tissue samples were stored in −80°C till further experiments. Whole RNA was extracted from five paired and eight orphan samples following TRIzol method followed by cDNA synthesis and Q-PCR analysis. Relative change in gene expression was normalized with 18SrRNA level. IHC was performed following the above-mentioned protocol with anti-FGF16 antibody (1: 50). All clinical and histo-pathological information were collected simultaneously. Collection/processing of tissue samples and follow-up experiments were done in SGCC&RI, Kolkata. The clinicopathological features of each of the clinical samples are mentioned in Supplementary Table S6.

### Statistical analysis

All data were calculated and analyzed using Microsoft excel/GraphPad Prism (Version 5.01) and expressed as mean ± SD of independent replicates of each experiment where ± SD was represented by error bars. Differences between treatments and controls were compared using one-way analysis of variance (ANOVA), followed by the post-hoc multiple comparisons—Tukey’s Honest Significant Test (HSD) whenever applicable (>2 experimental conditions), or using a Student’s *t*-test, otherwise (=2 experimental conditions). *P*<0.05 was considered as significant.

## Supplementary Material

Supplementary Figures S1-S5 and Tables S1-S6

## Data Availability

All primary data and constructs are freely available upon request to the corresponding author. The RNA-seq data will be deposited to the public repository during the process of revision.

## Abbreviations

2-NBDG: 2-NBD glucose
1D NMR: One-dimensional nuclear magnetic resonance
ATP: Adenosine triphosphate
BC: breast cancer
BSA: Bovine Serum Albumin
D_2_O: Deuterium oxide
DAPI: 4',6-diamidino-2-phenylindole
DMSO: Dimethylsulfoxide
ECM: extracellular matrix
EDTA: Ethylenediaminetetraacetic acid
EMT: epithelial–mesenchymal transition
FGF: fibroblast growth factor
GAPDH: glyceraldehyde-3 phosphate dehydrogenase
h: hour
MMP: matrix metalloproteinase
NaCl: sodium chloride
PBS: Phosphate buffered saline
PBST: Phosphate Buffered Saline with Tween 20
PMSF: phenyl methyl sulfonyl fluoride
RI: receptor inhibitor
SD: Standard deviation
shRNA: Short hairpin ribonucleic acid
siRNA: Small interfering ribonucleic acid
TCA: Tricarboxylic Acid
TSP: Trimethylsilylpropanoic acid
