# Supplementary material for: Reprogramming of glucose metabolism via PFKFB4 is critical in FGF16-driven invasion of breast cancer cells
Source: Biosci Rep. 2023 Aug 2;43(8):BSR20230677. doi: 10.1042/BSR20230677 (PMC10407156; doi:10.1042/BSR20230677)

Supplementary Material

Reprogramming of glucose metabolism via PFKFB4 is critical in FGF16-driven invasion of breast cancer cells

Swarnali Kar[1], Nilanjana Maji[1], Kamalika Sen[2], Stuti Roy[3], Atanu Maity[2], Shubhra Ghosh Dastidar[2], Somsubhra Nath[3, 4], Gautam Basu[1], Moitri Basu[1]

[1]Department of Biophysics, Bose Institute, P 1/12, C.I.T. Scheme VIIM, Kolkata- 700054, India.
[2]Bioinformatics Centre, Bose Institute, P 1/12, C.I.T. Scheme VIIM, Kolkata- 700054, India.

[3]Basic & Translational Research Division, Saroj Gupta Cancer Centre and Research Institute (SGCC & RI), Kolkata 700063, India

[4] Present address: Institute of Health Sciences, Presidency University, Kolkata 700156, India

[#] Address of corresponding author:
Dr. Moitri Basu, Department of Biophysics, Bose Institute, P 1/12, C.I.T. Scheme VIIM, Kolkata-700054, India. E-mail- Moitri_basu@jcbose.ac.in / moitrri.basu@gmail.com /gautam@jcbose.ac.in

Supplementary Methods

Molecular modeling

Structures of FGF16 in complex with any of the four FGFRs are not available. The complexes were modeled in two stages. First, the structure of FGF16-FGFR1 complex was homology modeled using FGF9-FGFR1 complex (PDB ID: 5w59) [1] as the template since FGF16 share significant amino acid sequence homology with FGF9 (Supplementary Figure 2). The modeling was carried out using Swiss-Model webserver [2]. FGFR1, FGFR2, FGFR3 and FGFR4 also share significant sequence similarity among themselves (Supplementary Figure 2). Hence, we used FGF16-FGFR1 as the template to model other three complexes i.e. FGF16-FGFR2, FGF16-FGFR3 and FGF16-FGFR4. The modeled structures were refined by energy minimization and by short molecular dynamics (MD) simulations.

Molecular Dynamics (MD) Simulation

Each of the modeled systems was solvated in a cubic water box of TIP3P [3] water molecules, ensuring at least 10Å solvent layer everywhere and maintaining K+ and Cl- ions sufficiently to meet the overall electrostatic neutrality and also 0.15 (M) salt concentration. Each system was energy minimized well and then was set to MD simulation. The initial 2 nanosecond (ns) was spent for heating the system to 300K and then to equilibrate under NVT condition at 1 atmosphere. It was followed by standard MD simulation for 5 ns under NPT condition. Temperature and pressure were controlled using a Nose-hover thermostat and barostat [4]. Coordinates were saved at an interval of 20 ps. The modeled systems were represented using CHARMM36 parameter set [5]. The modeling was done using CHARMM package [6] and then MD simulations were carried out in NAMD 2.12 [7].

Binding free energy calculation:

The binding free energy was calculated using the standard MMGBSA method [8-9]. Trajectories of FGF16, FGFR1/2/3/4 were extracted from the trajectory of FGF16-FGFR1/2/3/4. The potential energies of the systems were calculated in a continuum solvent to include the solvation energy terms. The binding free energy was calculated by subtracting energy of FGF16 and FGFR1/2/3/4 from that of the complex of FGF16 and FGFRi, where i=1, 2, 3 and 4 for different systems

$$\Delta G \text{ (Binding)} = G(\text{FGF16-FGFRiComplex}) - [\, G(\text{FGF16}) + G(\text{FGFRi}) \,]$$

Supplementary Results

Binding free energy calculation:

Using the modeled FGF16-FGFR1/2/3/4 structures, four separate 5 ns long MD simulations were performed that yielded ensemble representations of the complexes. The free energies of binding of each FGFR isoforms with FGF16 were then computed from the ensembles using the MMGBSA method, a very standard computational protocol for this purpose. Different components of interactions between the two proteins in each complex were obtained from this calculation as listed in Supplementary Table 1. It is widely accepted that for MMGBSA calculations, the absolute values are of lesser relevance; instead the relative order of the energies, i.e. ΔΔG is the significant index for making conclusions. The trend of ΔΔG indicates that although all the FGFRs have competitive affinities for FGF16, their subtle difference in affinities increases in the following order: FGR4<< FGFR2 < FGFR3 <FGFR1 ($\Delta\Delta G_{binding}$ listed in Supplementary Table 1).

Some representative snapshots were analyzed from the ensemble of the structures and specific interactions between FGF16 and FGFRs at their interface were checked. The analysis demonstrated that FGFR1 maintained favorable orientation of several charged residues that showed strong interaction with FGF16 which were absent for other FGFRs in the same position. For example, the orientation of D320 and K231 of FGFR1 and E95 and R107 of FGF16 are suitable for a pair of salt-bridge interaction between D320-R107 and K231-E95, all salt bridges. But this orientation was disrupted in other FGFRs either by reorientation of the residues or due to replacement by other residues. Similar alterations were also evident in other places of interactions, e.g. E159 and E162 from FGFR1 maintained salt-bridge interaction with R85 and K167 of FGF16, which were altered in other FGFRs leading to lowering in electrostatic interaction energy. Overall, these small differences have cumulatively contributed to the favorable interaction between FGF16 and FGFR1.

Analysis of sequence variation among FGFR isoforms at the binding interface with FGF16:

FGFR1 residues lying at the interface of FGF16-FGFR1 complex (PDB ID: 5w59) were identified by calculating the difference accessible surface area (ΔASA) of FGFR1 with and without the presence of FGF16 using DSSP webserver [http://bioinformatica.isa.cnr.it/SUSAN/DSSP-web/]. Any FGFR1 residue, associated with a non-zero value of ΔASA was considered to be at the interface. These residue positions were then highlighted in the multiple sequence alignment of the four FGFR isoforms (Supplementary Figure 2), which showed five sequence clusters at the binding interface (A-E). The 'EKxLxAVPAx**K**' motif of FGFR1 (where x indicates a non-interface residue) in cluster A was mostly conserved in all four isoforms, except the C-terminal 'K' that was replaced by 'N' in FGFR 2-4. The 'DxVxRxP' motif of FGFR1 in cluster B was mostly conserved in all four isoforms. The 'SD**P**QPH' motif of FGFR1 in cluster C was mostly conserved in all four isoforms, except the N-terminal 'P' which was replaced by 'A' in FGFR2-4. The 'GNSIxL' motif of FGFR1 in cluster E was mostly conserved in all four isoforms, except FGFR3 where the N-terminal 'GNS' stretch was replaced by 'TNF'. Marked differences were observed in cluster D: 'AGVxxTDKE' for FGFR1 and FGFR2, 'WISxxVEAD' for FGFR3 and 'ADIxxSE--' for FGFR4.

Supplementary References

1. Liu et al. (2017) Regulation of Receptor Binding Specificity of FGF9 by an Autoinhibitory Homodimerization. Structure, 25, 1325-1336 e3.

2. Waterhouse et al., (2018) SWISS-MODEL: homology modelling of protein structures and complexes. Nucleic Acids Res, 46(W1), W296-W303.

3. Jorgensen et al. (1983) Comparison of simple potential functions for simulating liquid water. J. Chem. Phys. 79, 926–935

4. Feller et al. (1995) Constant pressure molecular dynamics simulations: The Langevin piston method. J. Chem. Phys., 103, 4613– 4621.

5. Huang, J.; MacKerell, A. D., Jr. (2013) CHARMM36 all-atom additive protein force field: validation based on comparison to NMR data. J. Comput. Chem., 34, 2135– 2145.

6. Brooks, B.R., et al. (2009) CHARMM: the biomolecular simulation program. J. Comput. Chem., 30, 1545-614.

7. Phillips, J. C. et al (2005) Scalable molecular dynamics with NAMD. J. Comput. Chem., 26, 1781–1802.

8. Kollman et al. (2000) Calculating structures and free energies of complex molecules: combining molecular mechanics and continuum models Acc. Chem.Res., 33, 889−897

9. Swanson et al. (2004) Revisiting free energy calculations: a theoretical connection to MM/PBSA and direct calculation of the association free energy, Biophys. J., 86, 67−74.

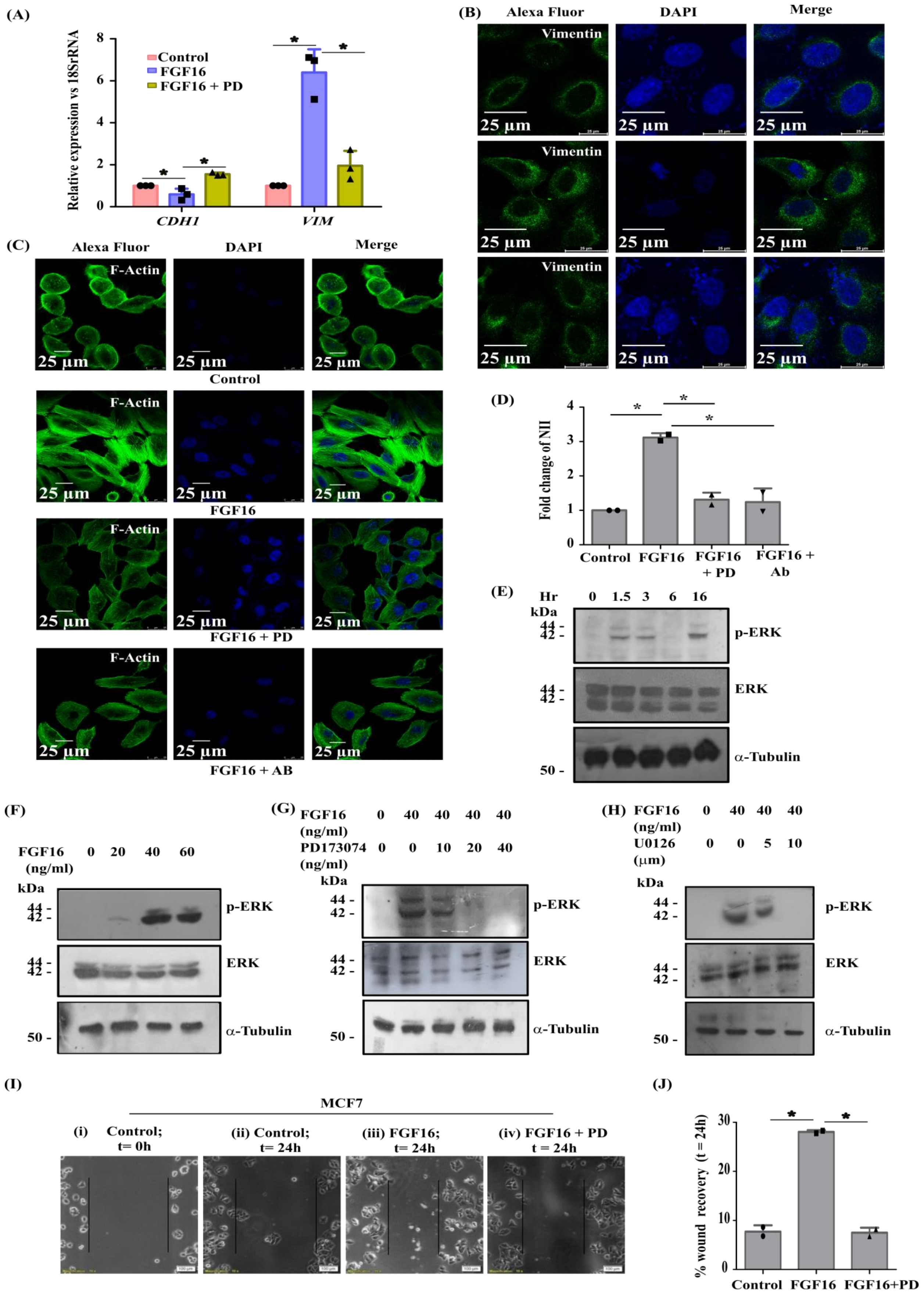

(A)
Relative expression vs 18SrRNA
Control
FGF16
FGF16 + PD
CDH1
VIM
(B)
Alexa Fluor
DAPI
Merge
Vimentin
25 µm
(C)
Alexa Fluor
DAPI
Merge
F-Actin
25 µm
Control
FGF16
FGF16 + PD
FGF16 + AB
(D)
Fold change of NII
Control
FGF16
FGF16 + PD
FGF16 + Ab
(E)
Hr
0
1.5
3
6
16
kDa
p-ERK
ERK
α-Tubulin
(F)
FGF16 (ng/ml)
0
20
40
60
(G)
FGF16 (ng/ml)
PD173074 (ng/ml)
(H)
FGF16 (ng/ml)
U0126 (µm)
(I)
MCF7
(i) Control; t= 0h
(ii) Control; t= 24h
(iii) FGF16; t= 24h
(iv) FGF16 + PD t = 24h
(J)
% wound recovery (t = 24h)
Control
FGF16
FGF16+PD

Supplementary Figure 1. (A) Quantitative determinations of EMT markers by Q-PCR assay was done with RNA isolated from cells, either untreated or treated with FGF16 alone or in combination with FGFRI. Relative gene expression is indicated as ‘fold’ change in the y-axis (mean ± SD). The statistical analysis was done by ANOVA followed by Tukey’s Honest Significant Test (HSD). * denotes $p < 0.05$ and ‘ns’ stands for non-significant changes. (B) Immunostaining with antibodies against Vimentin followed by Alexa Fluor-488 (green) and nuclei staining with DAPI was done in control (vehicle-treated) and FGF16-treated MCF10A cells. Magnification: 100x, Zoom Factor: 1.5, Scale bar: 25 µm. (C) Changes in actin organization were determined by immunostaining with Alexa Fluor 488 tagged-phalloidin in MCF10A cell-line after treatment with vehicle or FGF16 (alone or in combination with FGFRI or neutralizing antibody) for 24 h. The nuclei stained with DAPI and the merged images are also shown. Magnification: 63x, Zoom Factor: 1, Scale bar: 25 µm. (D) Nuclear irregularity index (NII) was calculated and the fold change with respect to control set was plotted for each treated set, considering DAPI images (n = 30) from two independent experimental sets using ImageJ with NMA plug-in as described. The statistical analysis was done by ANOVA followed by Tukey’s Honest Significant Test (HSD). * denotes $p < 0.05$ and ‘ns’ stands for non-significant changes. (E-H) Protein level of pMAPK and MAPK was assessed by Western blot with MCF10A cell lysate after respective treatment. (I) Wound healing assay was performed with MCF7 cells treated with FGF16 alone (iii) or in combination (iv) with PD in the time course of 24 h. t = 0 h at control cells in (i) signifies the time of scratching the cells. The boundary of the wound is marked by line which also indicates the width of wound. Scale bar: 100 µm. (J) The % recovery of the wound for each treated cells compared with control cells at 24 h was calculated and represented with statistical significance. The statistical analysis was done by ANOVA followed by Tukey’s Honest Significant Test (HSD). * denotes $p < 0.05$ and ‘ns’ stands for non-significant changes.

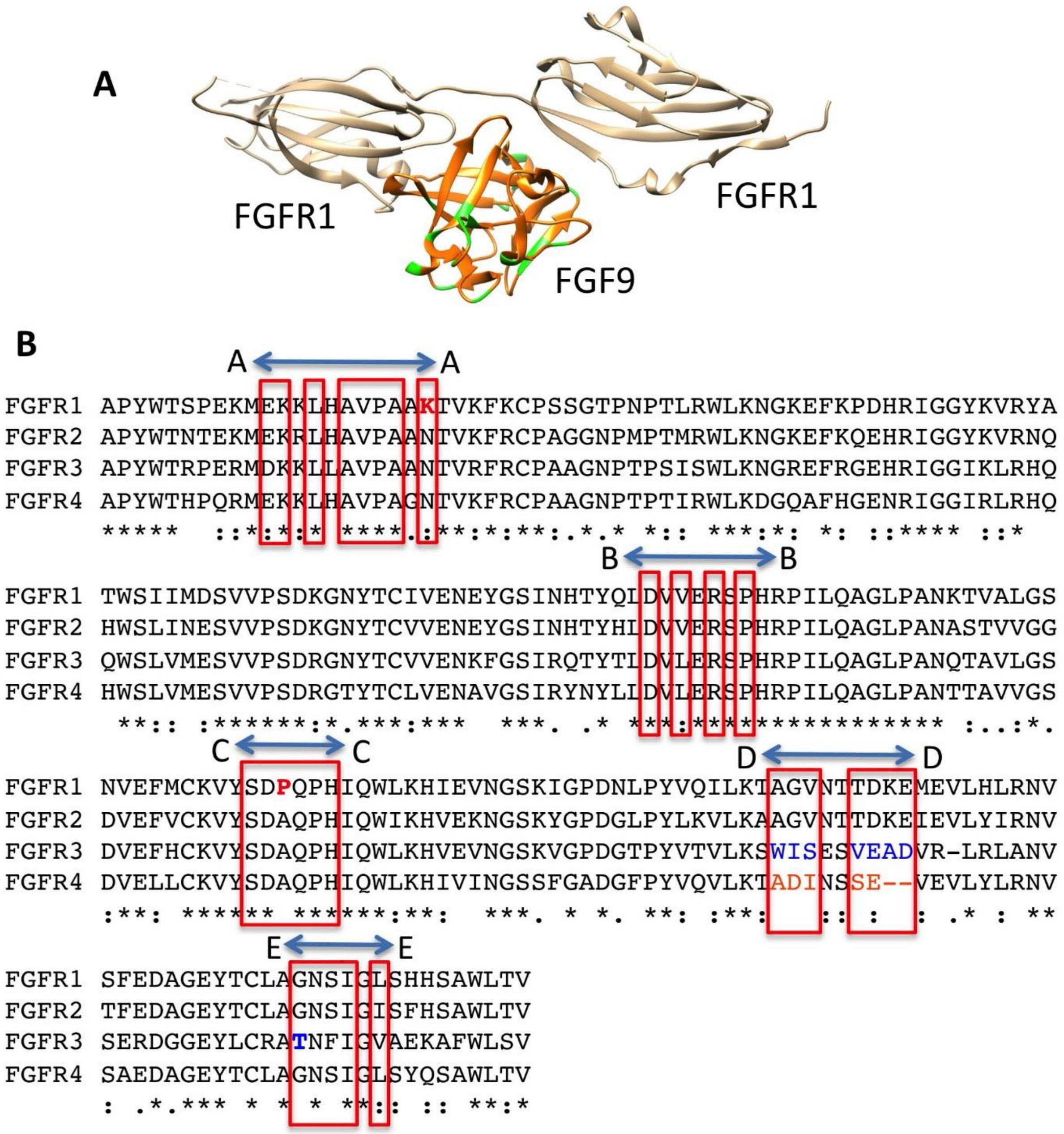

Supplementary Figure 2. (A) Cartoon representation of the crystal structure of FGF9-FGFR1 complex (PDB ID: 5w59). FGF9 is shown in orange color. Green colored residue positions differ between FGF16 and FGF9 (none at the interface with FGFR1) (B) Sequence alignment of FGFR1 (P11362|FGFR1_HUMAN), FGFR2 (P21802|FGFR2_HUMAN), FGFR3 (I6LM06|I6LM06_HUMAN) and FGFR4 (B5A965|B5A965_HUMAN). Square boxes represent sequence positions that lie at the interface between FGFR1 and FGF9 (in the structure shown in panel A). Clusters of boxed residues are annotated as A, B, C, D and E.

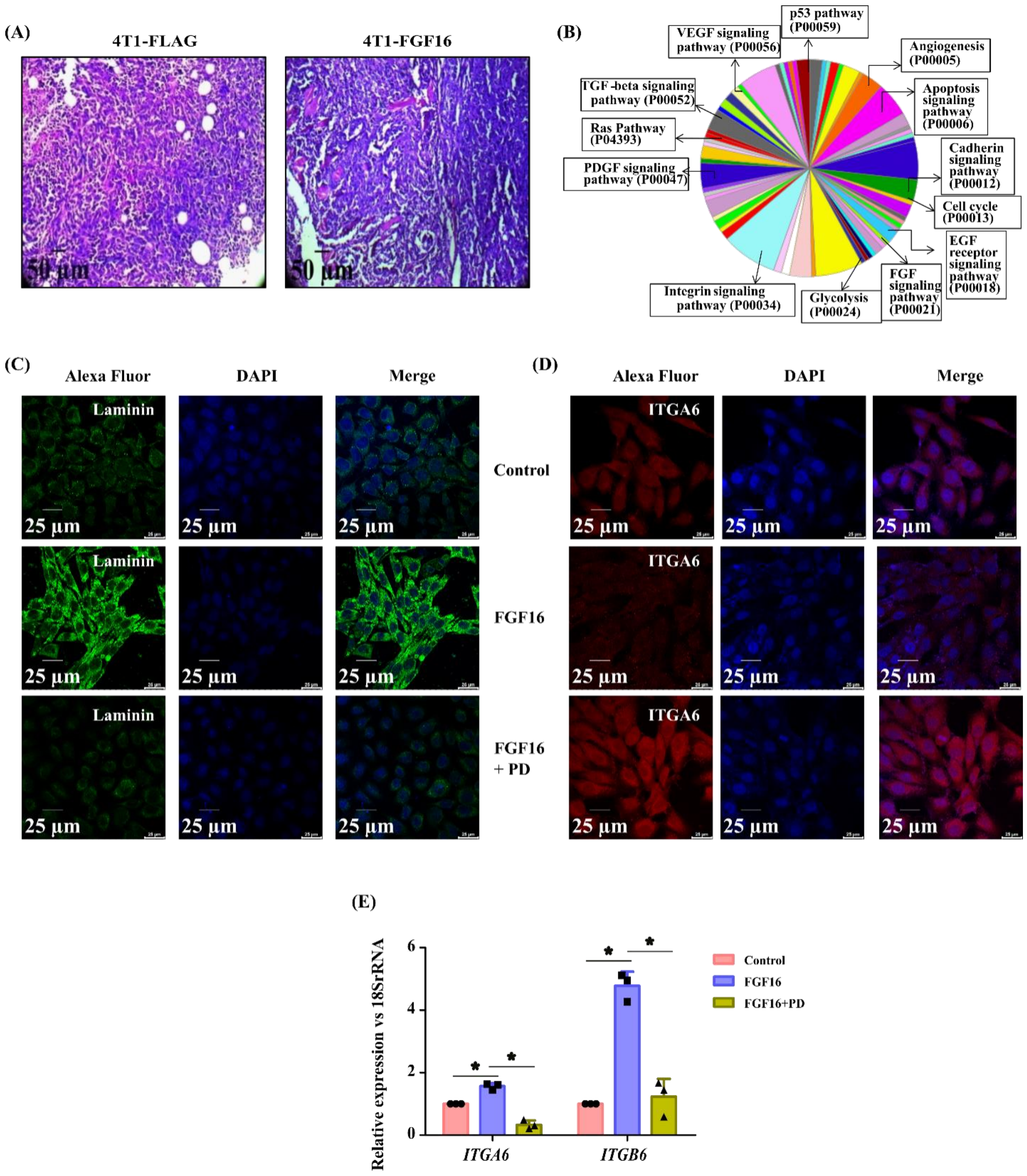

Supplementary Figure 3. (A) H-E staining was done with sections of 4T1-FLAG/4T1-FGF16-induced tumor tissue from mice. (B) The FGF16-induced up-regulated genes were analyzed by PANTHER classification system to point out the perturbed cellular pathways. (C-D) Immunostaining with antibodies against Laminin and Integrin A6 followed by Alexa Fluor-488 (green) and nuclei staining with DAPI were done in control (vehicle-treated) and FGF16-treated MCF10A cells. Magnification: 63X; Scale bar: 25µm. (E) Q-PCR assay was performed with RNA isolated from cells, treated as mentioned. Relative gene expression is indicated as ‘fold’ change in the y-axis (mean ± SD)

with statistical analysis by ANOVA followed by Tukey's Honest Significant Test (HSD). * denotes p < 0.05 and 'ns' stands for non-significant changes.

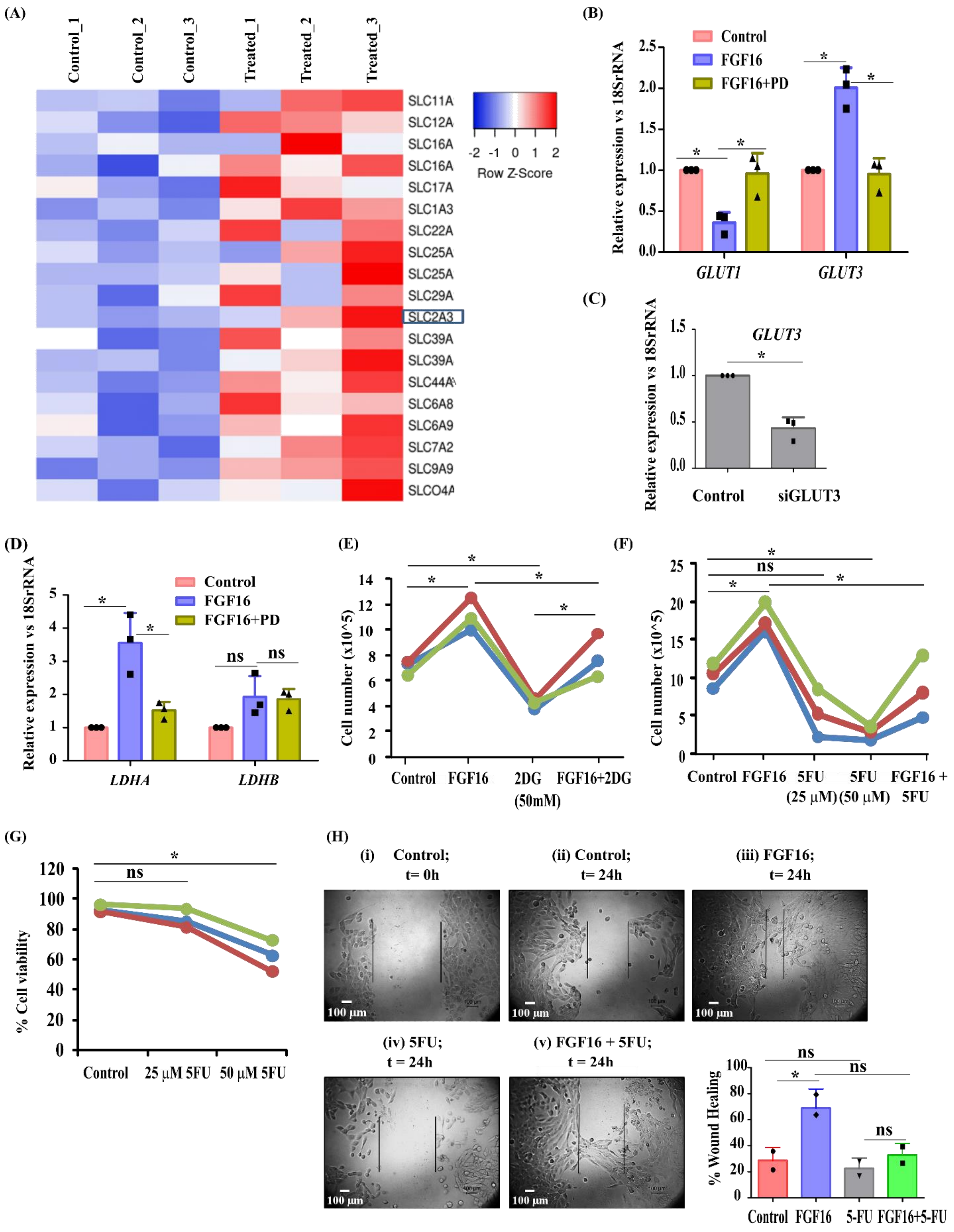

Supplementary Figure 4. (A) Heatmap showing differential expression signature of 19 genes from solute-carrier *gene* (*SLC*) super-family between control and FGF16-treated sets. The color code of expression values was also given. (B) Q-PCR assay was performed with RNA isolated from cells, treated as mentioned. Relative gene expression is indicated as 'fold' change in the y-axis (mean ± SD) with statistical analysis by ANOVA followed by Tukey's Honest Significant Test (HSD). * denotes $p < 0.05$ and 'ns' stands for non-significant changes. (C) The knockdown efficiency of si*GLUT3* was determined by Q-PCR assay. The statistical significance was calculated by two-tailed student's t-test. * denotes $p < 0.05$, 'ns' stands for non-significant change. (D) Quantitative determinations of *LDHA*, *LDHB* by Q-PCR assay was done with RNA isolated from cells, either untreated or treated with FGF16 alone or in combination with FGFRI. Relative gene expression is indicated as 'fold' change in the y-axis (mean ± SD) with statistical analysis by ANOVA followed by Tukey's Honest Significant Test (HSD). * denotes $p < 0.05$ and 'ns' stands for non-significant changes. (E-F) Cell numbers for mentioned sets were plotted in the y-axis (mean ± SD) with statistical analysis by ANOVA followed by Tukey's Honest Significant Test (HSD). * denotes $p < 0.05$ and 'ns' stands for non-significant changes. (G) Percent cell viability was calculated from Trypan Blue Assay and plotted in the y-axis (mean ± SD) with statistical analysis by ANOVA followed by Tukey's Honest Significant Test (HSD). * denotes $p < 0.05$ and 'ns' stands for non-significant changes. (H) Wound healing assay was performed with MCF10A cells treated with FGF16 alone (iii) or in combination (v) with 5-FU in the time course of 24 h. t = 0 h at control cells in (i) signifies the time of scratching the cells. The boundary of the wound is marked by line which also indicates the width of wound. Scale bar: 100 µm. The % recovery of the wound for each treated cells compared with control cells at 24 h was calculated and were plotted in the y-axis (mean ± SD) with statistical analysis by ANOVA followed by Tukey's Honest Significant Test (HSD). * denotes $p < 0.05$ and 'ns' stands for non-significant changes.

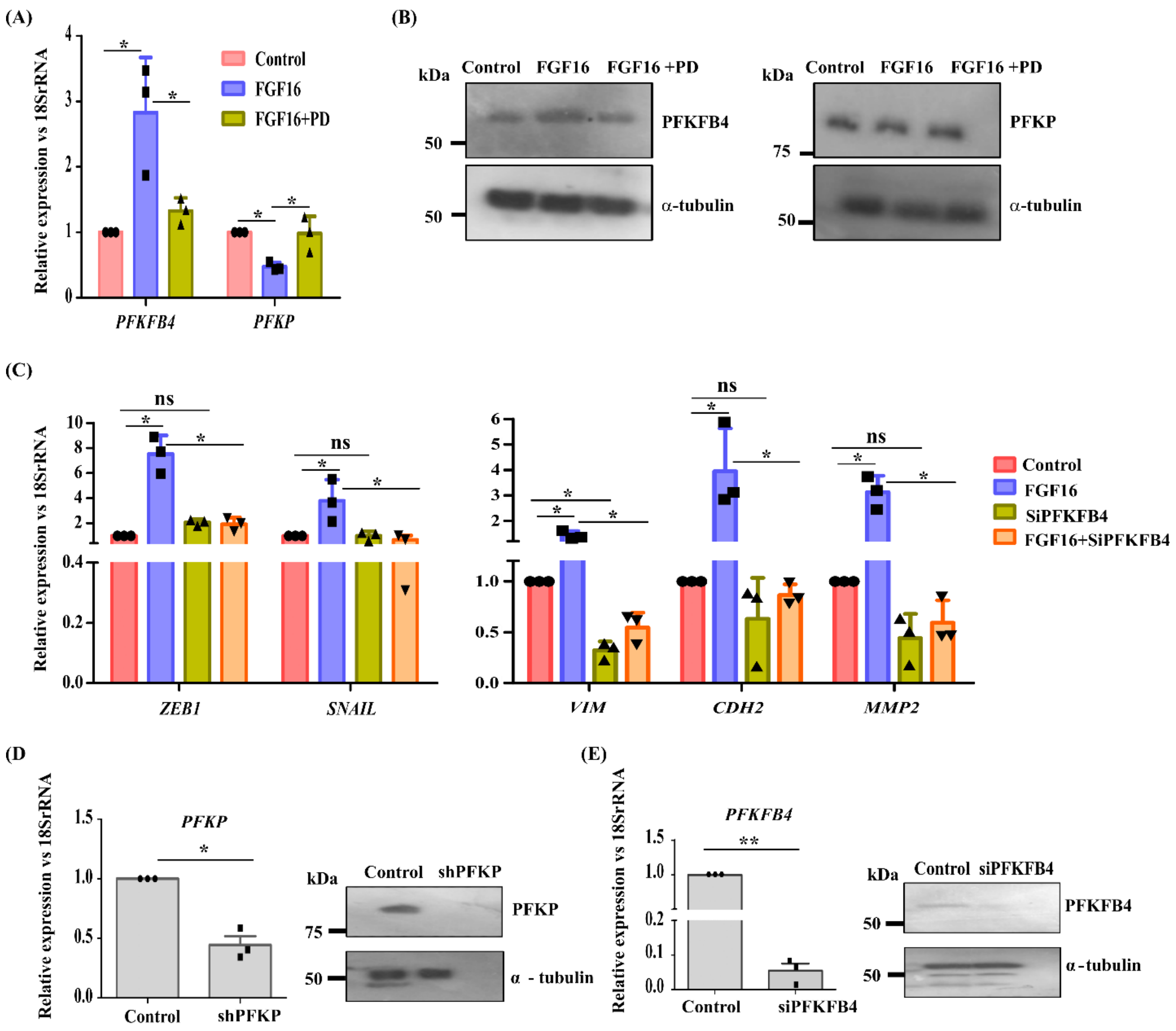

Supplementary Figure 5. (A) Quantitative determinations of *PFKP, PFKFB4* by Q-PCR assay was done with RNA isolated from cells, either untreated or treated with FGF16 alone or in combination with FGFRI. Relative gene expression is indicated as 'fold' change in the y-axis (mean ± SD) with statistical analysis by ANOVA followed by Tukey's Honest Significant Test (HSD). * denotes $p < 0.05$ and 'ns' stands for non-significant changes. (B) Protein level of PFKFB4 and PFKP was assessed by Western blot with MCF10A cell lysate after mentioned treatment. (C) Change in expression of *ZEB1, SNAIL, VIM, CDH2,* and *MMP2* were quantified by Q-PCR assay with RNA isolated from MCF10A cells. Relative gene expression is shown as 'fold' change in the y-axis (mean ± SD) with statistical analysis by ANOVA followed by Tukey's Honest Significant Test (HSD). * denotes $p < 0.05$, 'ns' stands for non-significant change. (D) The knockdown efficiency of sh*PFKP* was determined by Q-PCR assay and western blot. (E) The knockdown efficiency of si*PFKFB4* was determined by Q-PCR assay and Western blot. For D-E, The statistical significance was calculated by two-tailed student's t-test. * denotes $p < 0.05$, ** denotes $p < 0.005$ and 'ns' stands for non-significant change.

Supplementary Table 1: The binding (with FGF16) free energies (along with different components) of four FGFR isoforms. The energy values are in kcal/mol.

**Energy Components**	**FGFR1**	**FGFR2**	**FGFR3**	**FGFR4**
Electrostatic	-219.45	-174.19	-286.12	-105.89
Van der Waals	-102.65	-105.46	-109.94	-105.30
Internal energy	0.00	0.00	0.00	0.00
Polar solvation	223.31	184.26	299.28	119.35
Nonpolar solvation	-7.40	-7.61	-7.28	-7.39
Electrostatic + Polar solvation	3.81	10.10	13.19	13.49
Total	**-106.22**	**-102.99**	**-104.03**	**-99.15**
Relative Total	**0.0**	**2.2**	**3.2**	**7.1**

Supplementary Table 2-3

List of up-regulated and down-regulated genes from RNA-seq analysis.

**Supplementary Table 2. List of upregulated genes**			
**Gene name**	**Locus**	**Locus start**	**Locus end**
ZNF37BP	chr10	42513510	42552870
TLE1	chr9	81583682	81689535
HMGB3	chrX	1.51E+08	1.51E+08
DDX51	chr12	1.32E+08	1.32E+08
WHAMM	chr15	82809627	82834861
ANK2	chr4	1.13E+08	1.13E+08
PROS1	chr3	93873036	93974090
CENPF	chr1	2.15E+08	2.15E+08
ST3GAL1	chr8	1.33E+08	1.34E+08
HNRNPA3	chr2	1.77E+08	1.77E+08
LHFP	chr13	39342891	39603219
SLC39A11	chr17	72645945	73092714
ITGA4	chr2	1.81E+08	1.82E+08

**Supplementary Table 3. List of downregulated genes**			
**Gene name**	**Locus**	**Locus start**	**Locus end**
RN7SK	chr6	52995619	52995951
DMC1	chr22	38518948	38570196
RPPH1	chr14	20343070	20343411
RMRP	chr9	35657750	35658018
SCARNA2	chr1	1.09E+08	1.09E+08
HIST1H2AH	chr6	27147081	27147562
HIST2H3D	chr1	1.5E+08	1.5E+08
HIST1H2AM	chr6	27892698	27893185
MMP9	chr20	46008907	46016561
HIST1H2AG	chr6	27133037	27133535
HIST1H1E	chr6	26156330	26157115
RN7SL1	chr14	49586579	49586878
HIST1H2AI	chr6	27808198	27808667

TOP1MT	chr8	1.43E+08	1.43E+08
CDR2L	chr17	74987631	75005797
PSMB10	chr16	67934503	67936877
CUL9	chr6	43182183	43224587
SRRM2	chr16	2737075	2771412
PIF1	chr15	64815629	64825668
OSBPL5	chr11	3087115	3165352
ZBTB16	chr11	1.14E+08	1.14E+08
ANKFY1	chr17	4004444	4263979
IGF2R	chr6	1.6E+08	1.6E+08
GFPT2	chr5	1.8E+08	1.8E+08
IRX2	chr5	2746164	2751655
GCNT1	chr9	76441665	76507416
PDZD2	chr5	31798923	32110932
PIM3	chr22	49960494	49964072
MMP15	chr16	58025377	58046900
EVPL	chr17	76006845	76027426

HIST1H4E	chr6	26204644	26205021
RN7SL2	chr14	49862550	49862849
TERC	chr3	1.7E+08	1.7E+08
HIST2H2BF	chr1	1.5E+08	1.5E+08
HIST1H4H	chr6	26285125	26285499
TFPI2	chr7	93885396	93890991
HIST1H2BI	chr6	26272975	26273412
HIST1H2AJ	chr6	27814301	27814740
HIST1H2BF	chr6	26199558	26199988
HIST1H2BJ	chr6	27132315	27132796
HIST1H2BL	chr6	27807478	27807931
HIST1H4D	chr6	26188709	26189076
HIST4H4	chr12	14770719	14771131
SPRR2A	chr1	1.53E+08	1.53E+08
SBSN	chr19	35523366	35528351
HIST1H3C	chr6	26045410	26045869
SPRR1B	chr1	1.53E+08	1.53E+08

EHBP1L1	chr11	65576037	65592645
TENM4	chr11	78653282	79440651
UTP14A	chrX	1.3E+08	1.3E+08
TMEM63C	chr14	77181758	77259495
GRAMD4	chr22	46626760	46679791
TARBP1	chr1	2.34E+08	2.34E+08
TNIP1	chr5	1.51E+08	1.51E+08
KLF5	chr13	73054975	73077542
PARP6	chr15	72241180	72271287
PPARG	chr3	12287849	12434356
AMOTL2	chr3	1.34E+08	1.34E+08
UPF3A	chr13	1.14E+08	1.14E+08
TNS3	chr7	47275153	47582144
ZNF814	chr19	57869378	57889074
CCDC57	chr17	82101469	82212813
PSME1	chr14	24136157	24141588
ACACB	chr12	1.09E+08	1.09E+08

PPP1R14A	chr19	38251236	38256591
HIST1H2AE	chr6	26216919	26217483
HIST1H3H	chr6	27810063	27810536
HIST1H2AK	chr6	27837879	27838339
KRT14	chr17	41582278	41586895
HIST2H4A	chr1	1.5E+08	1.5E+08
ZG16B	chr16	2830171	2832284
PRSS3	chr9	33750465	33799231
HIST2H2AC	chr1	1.5E+08	1.5E+08
S100A7	chr1	1.53E+08	1.53E+08
HIST1H3J	chr6	27890314	27890792
HIST1H3F	chr6	26250141	26250607
LCE3D	chr1	1.53E+08	1.53E+08
SCG5	chr15_KI270905v1_alt	4856252	4911692
HIST1H4C	chr6	26103947	26104337
PPP2R2B	chr5	1.47E+08	1.47E+08
SPINK6	chr5	1.48E+08	1.48E+08

RBM6	chr3	49940043	50077252
PODXL	chr7	1.32E+08	1.32E+08
KLF13	chr15_KI270905v1_alt	3612678	3721509
HEG1	chr3	1.25E+08	1.25E+08
KLHL29	chr2	23385426	23708613
HNRNPD	chr4	82353313	82373996
FTL	chr19	48965308	48966879
SGSM2	chr17	2337511	2381054
NIPSNAP1	chr22	29554808	29581337
SUSD2	chr22	24181475	24189106
SDC3	chr1	30869465	30908633
RHBDF2	chr17	76470892	76501427
SAMD4A	chr14	54567611	54793315
PTGS2	chr1	1.87E+08	1.87E+08
PLD5	chr1	2.42E+08	2.43E+08
MITD1	chr2	99169262	99181029
JRK	chr8	1.43E+08	1.43E+08

ZNF460	chr19	57280484	57294068
LINC01605	chr8	37516405	37521386
LGALS7B	chr19	38789209	38791754
HIST1H2BH	chr6	26251650	26252075
HIST1H3G	chr6	26270917	26271384
DEPDC4	chr12	1E+08	1E+08
PI3	chr20	45174898	45176544
CHRNA9	chr4	40335328	40355217
ALOX5AP	chr13	30713477	30764428
SPRR2D	chr1	1.53E+08	1.53E+08
PTHLH	chr12	27958083	27971983
GPR68	chr14	91232531	91253880
HIST2H2AA3	chr1	1.5E+08	1.5E+08
INSC	chr11	15112423	15247208
HIST1H2BC	chr6	26123466	26123904
C11orf91	chr11	33698107	33700740
HIST1H3D	chr6	26196783	26199293

VEGFA	chr6	43770208	43786486
HDGFRP2	chr19	4472195	4517704
TRIM41	chr5	1.81E+08	1.81E+08
ANP32B	chr9	97983206	98015942
UTRN	chr6	1.44E+08	1.45E+08
MAN2A2	chr15	90904189	90922585
IRS2	chr13	1.1E+08	1.1E+08
AGPAT4	chr6	1.61E+08	1.61E+08
CD99L2	chrX	1.51E+08	1.51E+08
SLC12A8	chr3	1.25E+08	1.25E+08
CEBPD	chr8	47736913	47738164
EP300	chr22	41092609	41180077
TNFAIP3	chr6	1.38E+08	1.38E+08
TBL1X	chrX	9463294	9719740
TENM3	chr4	1.82E+08	1.83E+08
FBXW4	chr10	1.02E+08	1.02E+08
PAQR7	chr1	25861483	25871253

POMK	chr8	43093505	43123180
HIST1H2BG	chr6	26216200	26216644
ZNF724P	chr19	23223804	23250370
FUT3	chr19	5842887	5858239
GDPD3	chr16	30104809	30113557
CRLF1	chr19	18593224	18606850
MRGPRX3	chr11	18120954	18138480
ADAM19	chr5	1.57E+08	1.58E+08
TIMP1	chrX	47561099	47619857
SLPI	chr20	45252238	45254564
HIST1H2BD	chr6	26158120	26171348
IL11	chr19	55364381	55370463
LINC-PINT	chr7	1.31E+08	1.31E+08
TUBA1A	chr12	49184794	49189324
PWARSN,SNORD107	chr15	24981993	24983790
AFAP1L2	chr10	1.14E+08	1.14E+08
BCYRN1	chr2	47192404	47345074

ARRDC2	chr19	18001131	18014102
STRIP2	chr7	1.29E+08	1.29E+08
PRR15	chr7	29563810	29567295
TMEM156	chr4	38966745	39032421
ARHGEF4	chr2	1.31E+08	1.31E+08
HIRA	chr22	19330700	19431696
SLC1A3	chr5	36606354	36688334
ZCCHC2	chr18	62523424	62586743
AKAP17A	chrY	1591592	1643081
GOLM1	chr9	86026142	86100201
CASP1	chr11	1.05E+08	1.05E+08
PLK2	chr5	58453982	58460139
RAB13	chr1	1.54E+08	1.54E+08
BMP2	chr20	6768097	6780263
GOLGB1	chr3	1.22E+08	1.22E+08
KLF4	chr9	1.07E+08	1.07E+08
RHOB	chr2	20447070	20449443

KRT16	chr17	41609778	41612827
ITGB6	chr2	1.6E+08	1.6E+08
EPN3	chr17	50532686	50543750
TMCC3	chr12	94567123	94650562
TMEM191A	chr22	20701113	20704603
TLL1	chr4	1.66E+08	1.66E+08
BDKRB1	chr14	96256209	96264763
LPXN	chr11	58526870	58578239
TUBA4A	chr2	2.19E+08	2.19E+08
ANGPTL4	chr19	8364126	8374375
SESN3	chr11	95165512	95234404
LOC100129917	chr4	780148	781848
RPS18P9	chr6	1.5E+08	1.5E+08
SLC7A5P2	chr16	21517908	21520444
PPAPDC1A	chr10	1.2E+08	1.21E+08
PWAR5	chr15	24984859	24988232
ANKRD36BP1	chr1	1.68E+08	1.68E+08

NOP2	chr12	6556 869	656 833 2
IFI30	chr19	1817 3779	181 781 24
PIK3R1	chr5	6821 5755	683 018 21
SAV1	chr14	5063 3579	506 683 53
EVC2	chr4	5562 418	570 954 8
RRAGB	chrX	5571 7676	557 587 74
PRRC2C	chr1	1.71E +08	1.72 E+0 8
EIF3A	chr10	1.19E +08	1.19 E+0 8
LEO1	chr15	5193 8024	519 718 01
COL4A5	chrX	1.08E +08	1.09 E+0 8
FYCO1	chr3	4591 7898	459 958 24
KRI1	chr19	1054 3893	105 660 26
SEMA4C	chr2	9685 9735	968 699 98
MAN1A1	chr6	1.19E +08	1.19 E+0 8
MAGI1	chr3	6535 4230	660 388 34
PAM	chr5	1.03E +08	1.03 E+0 8
NTN4	chr12	9564 9254	957 907 58

PLEK2	chr14	6738 6982	6741 2111
LOC1002887 98	chr12	4638 3675	4665 2579
HSF2BP	chr21	4352 9191	4365 9493
EAF2	chr3	1.22E +08	1.22E +08
DAAM1	chr14	5918 8662	5937 1405
HIST2H2BE	chr1	1.5E+ 08	1.5E+ 08
S100P	chr4	6693 838	6697 170
RPL13AP20	chr12	1287 5476	1287 6136
WNT7A	chr3	1381 8584	1388 0121
PMEPA1	chr20	5764 8391	5771 2780
HIST1H1C	chr6	2605 5739	2605 6471
PCSK1N	chrX	4883 1091	4883 5638
LINC01133	chr1	1.6E+ 08	1.6E+ 08
RASSF6	chr4	7357 1549	7362 0631
ICAM3	chr19	1033 3775	1033 9669
PFN1P2	chr1	1.2E+ 08	1.2E+ 08
MPP7	chr10	2805 0993	2828 2138

DDIT4	chr10	72273918	72276039
FLOT1	chr6_GL000256v2_alt	2028519	2043472
NFE2L1	chr17	48048323	48061545
MGLL	chr3	1.28E+08	1.28E+08
GPATCH4	chr1	1.57E+08	1.57E+08
ACIN1	chr14	23058564	23100456
ARHGEF16	chr1	3454582	3481113
ITGA10	chr1	1.46E+08	1.46E+08
IL1R1	chr2	1.02E+08	1.02E+08
ADARB1	chr21	45074577	45226563
ZNF692	chr1	2.49E+08	2.49E+08
DAPK1	chr9	87497227	87708634
AFAP1L1	chr5	1.49E+08	1.49E+08
WWC1	chr5	1.68E+08	1.68E+08
SGPP2	chr2	2.22E+08	2.23E+08
PLEKHA6	chr1	2.04E+08	2.04E+08
KIAA0922	chr4	1.53E+08	1.54E+08

COL6A3	chr2	2.37E+08	2.37E+08
DOK7	chr4	3463305	3501473
LINC01004	chr7	1.05E+08	1.05E+08
TMCC2	chr1	2.05E+08	2.05E+08
FAM218A	chr4	1.65E+08	1.65E+08
S100A2	chr1	1.54E+08	1.54E+08
MYEOV	chr11	69294137	69297287
EPHB2	chr1	22710837	22921500
C8orf48	chr8	13566842	13568288
PLAU	chr10	73909968	73922777
TGM4	chr3	44874605	44914596
NIPAL4	chr5	1.57E+08	1.57E+08
SH2D2A	chr1	1.57E+08	1.57E+08
LAMC2	chr1	1.83E+08	1.83E+08
NT5DC2	chr3	52495339	52535077
ANXA8L1	chr10	46375589	46391783
LAT2	chr7	74209756	74229834

ZNF362	chr1	33256572	33300719
WARS	chr14	1E+08	1E+08
SEL1L3	chr4	25747426	25863595
GPR37	chr7	1.25E+08	1.25E+08
PRPF40B	chr12	49623413	49707414
ERP29	chr12	1.12E+08	1.12E+08
WDR60	chr7	1.59E+08	1.59E+08
C10orf2	chr10	1.01E+08	1.01E+08
ZNF395	chr8	28345584	28386460
CLCN4	chrX	10156944	10237659
LONRF1	chr8	12721896	12755483
PSME2	chr14	24143364	24146646
NFIL3	chr9	91409044	91424626
TM4SF1	chr3	1.49E+08	1.49E+08
EGFR	chr7	55019031	55207338
ANKRD11	chr16	89267620	89490561
TRAF3IP1	chr2	2.38E+08	2.38E+08
STARD8	chrX	6864	687

AMIGO2	chr12	47075706	47236663
ALOXE3	chr17	8095899	8118916
NUAK2	chr1	2.05E+08	2.05E+08
PRADC1	chr2	73228005	73233228
SERPINB5	chr18	63476910	63505085
MIR205HG	chr1	2.09E+08	2.09E+08
ADTRP	chr6	11713654	11779047
SLC46A3	chr13	28700080	28719013
RAB7B	chr1	2.06E+08	2.06E+08
P2RY2	chr11	73218297	73242427
SERPINE1	chr7	1.01E+08	1.01E+08
ADORA1	chr1	2.03E+08	2.03E+08
ZNF121	chr19	9565727	9584533
NAA38	chr17	7856684	7862282
LOC100287015	chr8	6403555	6406548
C7orf13	chr7	1.57E+08	1.57E+08
TUBB3	chr16	89922008	89936097
CCDC126	chr7	2359	2364

		7668	25842
MZF1	chr19	58559185	58574797
HEXIM1	chr17	45147316	45152101
CLK2	chr1_GL383519v1_alt	67890	78552
STAT4	chr2	1.91E+08	1.91E+08
ZC3HAV1	chr7	1.39E+08	1.39E+08
ACACA	chr17_KI270857v1_alt	1320991	1645967
NPC1	chr18	23503469	23586617
TMEM144	chr4	1.58E+08	1.58E+08
MC1R	chr16	89917878	89920977
COL4A1	chr13	1.1E+08	1.1E+08
TUBGCP6	chr22	50217688	50244971
PKD1	chr16	2047988	2135898
LDLR	chr19	11089361	11133829
SLC17A5	chr6	73593378	73654014
PCYOX1L	chr5	1.49E+08	1.49E+08
PLAC8	chr4	83090057	83114758
B4GALNT4	chr11	369794	382117

		7378	4708
TNC	chr9	1.15E+08	1.15E+08
SEPW1	chr19	47778584	47784686
OSGIN1	chr16	83953221	83966332
COL1A1	chr17	50184095	50201639
LOC284454	chr19	13834515	13836359
EVA1B	chr1	36322029	36324154
STON2	chr14	81260649	81427404
LOC644656	chr11	9459555	9460698
JOSD2	chr19	50505996	50511355
SEMA7A	chr15	74409288	74433958
C1orf106	chr1	2.01E+08	2.01E+08
PTPRZ1	chr7	1.22E+08	1.22E+08
FUT1	chr19	48748010	48755390
LFNG	chr7	2512528	2529176
ZNF808	chr19	52527655	52556050
KMT2E-AS1	chr7	1.05E+08	1.05E+08
GK	chrX	30653358	30731460

PIGZ	chr3	1.97E+08	1.97E+08
HNRNPU	chr1	2.45E+08	2.45E+08
ABTB1	chr3	1.28E+08	1.28E+08
SLC6A9	chr1	43991607	44031492
MGEA5	chr10	1.02E+08	1.02E+08
CLMN	chr14	95181938	95319908
FBN2	chr5	1.28E+08	1.29E+08
TRIM38	chr6	25962688	25987329
HNRNPA2B1	chr7	26189935	26200793
HERC2P9	chr15_KI270905v1_alt	865220	961068
IFT172	chr2	27442365	27489811
RBM43	chr2	1.51E+08	1.51E+08
LINC01000	chr7	1.29E+08	1.29E+08
BAHCC1	chr17	81399720	81466332
MEGF8	chr19	42325608	42378769
LAMC1	chr1	1.83E+08	1.83E+08
ZNF251	chr8_KI270815v1_alt	95391	130068
TP53	chr17	7668401	7770350

CSF1R	chr5	1.5E+08	1.5E+08
DENND2C	chr1	1.15E+08	1.15E+08
ZBTB26	chr9	1.23E+08	1.23E+08
RAB30-AS1	chr11	83072065	83073712
ACAA2	chr18	49783503	49813881
LYPD3	chr19	43460793	43465679
RHOD	chr11	67056817	67072017
DYNC1I1	chr7	95772505	96110322
FAM213A	chr10	80407828	80432997
DLX2	chr2	1.72E+08	1.72E+08
MLLT11	chr1	1.51E+08	1.51E+08
ADGRF4	chr6	47698552	47722021
PARD6B	chr20	50731543	50753741
NXN	chr17	799312	979758
HOMER2	chr15	82848976	82952724
LINC01503	chr9	1.29E+08	1.29E+08
CMTM7	chr3	32391670	32454841
FAM212A	chr3	49803253	49805030

			2
MAFF	chr22	38201931	38216510
GALK1	chr17	75757936	75765199
AGRN	chr1	1020122	1056119
MLLT6	chr17_KI270857v1_alt	2740938	2765122
ANKRD18A	chr9	38571363	38620363
OMA1	chr1	58480718	58546799
KCNK5	chr6	39188970	39229475
ALDH3B1	chr11	68008546	68029282
RPS6KA2	chr6	1.66E+08	1.67E+08
SNX8	chr7	2251769	2314475
ZNF165	chr6	28080703	28089562
TCEAL4	chrX	1.04E+08	1.04E+08
MAOA	chrX	43654906	43746824
BBS12	chr4	1.23E+08	1.23E+08
RERG	chr12	15107781	15221477
FOXO3	chr6	1.09E+08	1.09E+08
MORC2	chr22	30922307	309684

RNF125	chr18	32018481	32073191
DDB2	chr11	47214941	47239218
NOL4L	chr20	32443058	32585073
IRAK1BP1	chr6	78867543	78900966
SUV420H2	chr19	55339852	55348121
RPL39	chrX	1.2E+08	1.2E+08
C19orf70	chr19	5678413	5688523
PFDN4	chr20	54207962	54219953
TEFM	chr17	30898982	30906268
RPS29	chr14	49576671	49586416
SELK	chr3	53885198	53891962
CSTA	chr3	1.22E+08	1.22E+08
GPM6B	chrX	13770942	13938712
BTBD11	chr12	1.07E+08	1.08E+08
UBA6-AS1	chr4	67701277	67722504
PXMP4	chr20	33702743	33720330
GPAM	chr10	1.12E+08	1.12E+08

			97
PTPRM	chr18	7567315	8406861
ZC3H13	chr13	45962178	46052761
KCNIP3	chr2	95297323	95386077
ZNF274	chr19	58182988	58213562
PFDN6	chr6_GL000255v2_alt	4484020	4485358
KRT15	chr17	41513744	41519018
GOLGA8B	chr15	34525282	34583570
COL4A6	chrX	1.08E+08	1.08E+08
TAP2	chr6_GL000256v2_alt	4221234	4238073
OPTN	chr10	13100081	13138276
TPR	chr1	1.86E+08	1.86E+08
GLI4	chr8	1.43E+08	1.43E+08
CYR61	chr1	85580760	85583965
P4HTM	chr3	48989907	49007148
GPC6	chr13	93226824	94408019
PML	chr15	73994672	74047814
PNISR	chr6	99399964	994313

TRAPPC5	chr19	7680820	7682862
TMTC3	chr12	88142295	88199887
PARD6G	chr18	80147923	80247514
CKMT1B	chr15	43593053	43599406
KRT6B	chr12	52446650	52452126
ZBED2	chr3	1.12E+08	1.12E+08
COX7C	chr5	86617966	86620766
MAP3K7CL	chr21	29077470	29175889
RELL1	chr4	37453929	37686377
HIST1H2AC	chr6	26124144	26124690
C19orf33	chr19	38303559	38315966
TUBB2B	chr6	3224260	3227734
ACOT11	chr1	54548133	54634744
TXNDC9	chr2	99319023	99336397
ANKRD39	chr2	96847986	96858019
TXNDC17	chr17	6578324	6651634
SPC24	chr19	11147154	11155808

			14
CNTRL	chr9	1.21E+08	1.21E+08
MSL3P1	chr2	2.34E+08	2.34E+08
LOC100288778	chr12_GL877875v1_alt	4476	22015
TLE4	chr9	79571772	79726881
MVD	chr16	88651939	88663149
GOLGA2	chr9	1.28E+08	1.28E+08
C19orf68	chr19	48170691	48197620
SULT1E1	chr4	69841211	69860152
FZD2	chr17	44557443	44561262
SLFN12	chr17	35410921	35433176
AGAP6	chr10	49988317	50010499
CACNB1	chr17	39173455	39197703
COBL	chr7	51016211	51316818
PNPLA3	chr22	43923738	43947568
MOV10	chr1	1.13E+08	1.13E+08
CRIM1	chr2	36356226	36551135
COL4A2	chr13	1.1E+08	1.11E+08

BRI3BP	chr12	1.25E+08	1.25E+08
FAM111B	chr11	59107184	59127416
DAW1	chr2	2.28E+08	2.28E+08
RPL22L1	chr3	1.71E+08	1.71E+08
HAT1	chr2	1.72E+08	1.72E+08
RPL9	chr4	39454124	39458948
ZBED8	chr5	1.6E+08	1.6E+08
IRGQ	chr19	43584366	43596135
RAB38	chr11	88113246	88175467
C16orf74	chr16	85707517	85751083
AP1M2	chr19	10572670	10587315
TP53I3	chr2	24076873	24169638
TPRKB	chr2	73729829	73737390
RPL34	chr4	1.09E+08	1.09E+08
CKMT1A	chr15	43692885	43699222
RFTN1	chr3	16315844	16513715
SNRPG	chr2	70281373	70293737

PNRC1	chr6	89080709	89085160
PNPT1	chr2	55634062	55693910
PDLIM2	chr8	22578740	22598025
CROCCP2	chr1	16618255	16630906
MYD88	chr3	38138477	38143021
CD68	chr17	7579486	7582111
GIGYF1	chr7	1.01E+08	1.01E+08
RRS1	chr8	66429027	66430733
SAFB	chr19	5623034	5668478
CD274	chr9	5450502	5470567
SLTM	chr15	58879044	58933653
NRCAM	chr7	1.08E+08	1.08E+08
LZTS2	chr10	1.01E+08	1.01E+08
C10orf54	chr10	71396933	71815947
TPRA1	chr3	1.28E+08	1.28E+08
TSC22D3	chrX	1.08E+08	1.08E+08
SRSF4	chr1	29147737	29182125

USMG5	chr10	1.03E+08	1.03E+08
ELAVL2	chr9	23690104	23826065
SVIP	chr11	22822051	22829836
ZMAT5	chr22	29720354	29766980
TAX1BP3	chr17	3636467	3696404
CDK2AP2	chr11	67506489	67508728
OVCA2	chr17	2030136	2043431
TUBB2A	chr6	3153665	3157549
HBEGF	chr5	1.4E+08	1.4E+08
SLC25A20	chr3	48856922	48898993
MMP28	chr17_KI270857v1_alt	388	30239
CD24	chr6	1.07E+08	1.07E+08
RNFT1	chr17	59952361	59964756
MTURN	chr7	30134935	30162765
RPS21	chr20	62387064	62388520
OBFC1	chr10	1.04E+08	1.04E+08
CCDC167	chr6	37482920	37499924

ALDH6A1	chr14	74019355	74084493
LOC146880	chr17	64749661	64781999
TRIM66	chr11	8612036	8658836
HABP4	chr9	96450154	96491336
SLC16A5	chr17	75087726	75106160
FKBP5	chr6	35573584	35736947
SH3TC1	chr4	8199332	8241103
SHANK3	chr22	50674641	50733212
CFDP1	chr16	75293709	75433489
TCEAL3	chrX	1.04E+08	1.04E+08
NT5C3A	chr7	33014112	33062797
LAMB1	chr7	1.08E+08	1.08E+08
ARID5B	chr10	61901253	62096948
LOC642852	chr21	45288051	45297354
NLGN2	chr17	7408182	7419864
RBCK1	chr20	408064	430966
PPP1R15A	chr19	48872391	48876062
PTX3	chr3	1.57E	1.58

ATP13A2	chr1	16985957	17011972
SH3BGRL3	chr1	26279721	26281522
TRIM16L	chr17	18722088	18736118
RPS19BP1	chr22	39529092	39532855
POLR2K	chr8	1E+08	1E+08
RAC2	chr22	37225260	37244299
AKR1C1	chr10	4963261	4977966
MAD2L1	chr4	1.2E+08	1.2E+08
PRICKLE2	chr3	64067963	64225455
TTC33	chr5	40711575	40755970
KLHL25	chr15	85759325	85794958
LIN7C	chr11	27494417	27506779

		+08	E+0 8
ALDH5A1	chr6	2449 4968	245 372 07
TRIM25	chr17	5688 7908	569 140 48
VSTM2L	chr20	3790 3096	379 453 45
NUP210	chr3	1331 6229	134 203 19
LMO7	chr13	7554 9479	758 598 71
LOC100288 637	chr15_KI2709 05v1_alt	3768 956	397 462 9
NRARP	chr9	1.37E +08	1.37 E+0 8
JDP2	chr14	7542 7805	754 727 01
BIRC3	chr11	1.02E +08	1.02 E+0 8
PXYLP1	chr3	1.41E +08	1.41 E+0 8
C1orf115	chr1	2.21E +08	2.21 E+0 8
TNFAIP2	chr14	1.03E +08	1.03 E+0 8
FAM46A	chr6	8174 5729	817 527 11
TMEM171	chr5	7312 0560	731 318 17
PCNT	chr21	4632 4121	464 457 69
NAT8L	chr4	2059 511	206 908 9
PRKAG2	chr7	1.52E	1.52

		+08	E+08
HDAC5	chr17	44076752	44123646
IL18R1	chr2	1.02E+08	1.02E+08
IRF1	chr5	1.32E+08	1.32E+08
NCL	chr2	2.31E+08	2.31E+08
IL22RA1	chr1	24119770	24143285
HNRNPUL2	chr11	62690261	62727385
STC2	chr5	1.73E+08	1.73E+08
BCL6	chr3	1.88E+08	1.88E+08
CHD2	chr15	92900320	93028007
FTSJ1	chrX	48476020	48486364
SOCS1	chr16	11254416	11256182
AMH	chr19	2249113	2252073
MEGF6	chr1	3487941	3611495
PABPN1	chr14	23306761	23326185
LRRC37B	chr17	32021135	32053501
PRDM8	chr4	80185269	80204328
SRSF5	chr14	6976	697

		6282	72005
CDK11A	chr1	1702730	1724352
VASH1	chr14	76761891	76786724
MT1E	chr16	56625672	56627113
THSD4	chr15	71141448	71783383
CCDC69	chr5	1.51E+08	1.51E+08
KCNAB2	chr1	5862807	6101193
OPN3	chr1	2.42E+08	2.42E+08
GAR1	chr4	1.1E+08	1.1E+08
UHRF1	chr19	4903079	4962154
ZNF300	chr5	1.51E+08	1.51E+08
LENG8	chr19_KI270938v1_alt	431453	446008
ICAM1	chr19	10270840	10286615
SLC25A28	chr10	99610517	99620464
HDX	chrX	84317873	84502479
HERC2P3	chr15	20408395	20506180
PLA2G6	chr22	38111494	38181829
SP100	chr2	2.3E+08	2.31E+0

			8
FUCA1	chr1	2384 5080	238 683 69
CITED2	chr6	1.39E +08	1.39 E+0 8
SPIRE2	chr16	8982 8498	898 713 19
FOSL2	chr2	2838 4408	284 146 49
PPAP2B	chr1	5649 4746	565 795 84
HERC2P2	chr15_KI2709 05v1_alt	8652 20	961 068
TRIM5	chr11	5663 194	568 510 9
R3HDM2	chr12	5725 3764	573 104 63
IGFBP6	chr12	5309 7651	531 023 44
CMTR1	chr6	3743 3130	374 815 08
FBN1	chr15	4840 8305	486 457 88
TAF15	chr17_KI2708 57v1_alt	4398 6	817 74
COL6A1	chr21	4598 1748	460 050 49
SEMA6B	chr19	4542 587	455 975 9
SYNE2	chr14	6385 2964	642 264 51
GDA	chr9	7211 4594	722 522 24
WASH3P	chr15	1.02E +08	1.02 E+0 8
PARP12	chr7	1.4E+	1.4

		08	E+08
HLA-E	chr6_GL000256v2_alt	1790174	1794974
SH3RF3	chr2	1.09E+08	1.1E+08
SCNN1G	chr16	23182718	23216879
TXNRD1	chr12	1.04E+08	1.04E+08
KLF9	chr9	70384596	70414657
CFH	chr1	1.97E+08	1.97E+08
MYLIP	chr6	16129085	16148247
CXCL1	chr4	73869391	73871302
GOLGA8A	chr15	34379068	34437466
IFIT5	chr10	89414567	89421002
LIMCH1	chr4	41359606	41700044
SDSL	chr12	1.13E+08	1.13E+08
NEXN	chr1	77881347	77943893
LSS	chr21_KI270872v1_alt	49815	80944
ALDOC	chr17	28573114	28576933
CARD16	chr11	1.05E+08	1.05E+08
ABCD1	chrX	1.54E+08	1.54E+0

			8
FMNL1	chr17	4522 1924	452 473 16
PBXIP1	chr1	1.55E +08	1.55 E+0 8
PABPC1L	chr20	4491 0061	449 393 21
OGFR	chr20	6280 0626	628 140 00
PNN	chr14	3917 5182	391 832 18
SYNGAP1	chr6_GL0002 56v2_alt	4868 794	490 241 2
CCDC71L	chr7	1.07E +08	1.07 E+0 8
RILPL1	chr12	1.23E +08	1.24 E+0 8
MAP2	chr2	2.09E +08	2.1 E+0 8
GAS1	chr9	8694 4361	869 471 89
CYP4X1	chr1	4702 3567	470 507 51
FBLN5	chr14	9186 9410	919 477 02
MT1X	chr16	5668 2469	566 841 96
ACSL3	chr2	2.23E +08	2.23 E+0 8
ZNF862	chr7	1.5E+ 08	1.5 E+0 8
STC1	chr8	2384 1920	238 548 07
BTN3A2	chr6	2636 5158	263 783

			20
SLC7A2	chr8	17497087	17570568
POU5F1	chr6_GL000256v2_alt	2467752	2474125
ERRFI1	chr1	8011718	8026333
WASH1	chr9	11986	29739
VIPR1	chr3	42489298	42537573
PCDH7	chr4	30720407	31146801
FAM95C	chr9	38540566	38545372
FBXO32	chr8	1.23E+08	1.24E+08
SOD2	chr6	1.6E+08	1.6E+08
SVEP1	chr9	1.1E+08	1.11E+08
AASS	chr7	1.22E+08	1.22E+08
PFKFB4	chr3	48517683	48556794
LOXL2	chr8	23296896	23404209
EMG1	chr12	6970780	6976003
C22orf46	chr22	41690542	41698136
PATL1	chr11	59636715	59669038
LAMA5	chr20	62309059	62367312

LOC729218	chr4	1.19E+08	1.19E+08
FAAH2	chrX	57286676	57489196
IL1RN	chr2	1.13E+08	1.13E+08
CLSTN3	chr12	7130370	7158934
TCEA2	chr20	64057085	64072347
PEG10	chr7	94656324	94669694
NAMPT	chr7	1.06E+08	1.06E+08
HOOK2	chr19	12763002	12775620
GRTP1	chr13	1.13E+08	1.13E+08
DKK1	chr10	52314280	52317657
N4BP1	chr16	48538725	48610209
HMOX1	chr22	35381066	35394214
C14orf159	chr14	91114012	91225396
FASN	chr17	82078337	82098230
LY6E	chr8_KI270817v1_alt	8772	12698
SLIT2	chr4	20253563	20620561
CNTN3	chr3	74262568	74521140
NPIPB3	chr16	2140	214

		2133	25337
AGAP9	chr10	47501853	47523638
NNMT	chr11	1.14E+08	1.14E+08
CSF1	chr1	1.1E+08	1.1E+08
WFDC21P	chr17	60083565	60088467
NPIPB5	chr16	22513522	22536520
GRIPAP1	chrX	48973719	49002264
A4GALT	chr22	42692120	42720870
GTF2IP20	chr1	2.24E+08	2.24E+08
CXCL2	chr4	74097036	74099280
SDPR	chr2	1.92E+08	1.92E+08
GSDMD	chr8_KI270816v1_alt	43426	53101
HMGCS1	chr5	43287469	43313512
GOLGA2P5	chr12	1E+08	1E+08
SREBF1	chr17	17681472	17837011
ALPP	chr2	2.32E+08	2.32E+08
IL7R	chr5	35856874	35879603
SLC39A8	chr4	1.02E+08	1.02E+08

UNC93B1	chr11	67991099	68004125
C1QTNF1	chr17	79019208	79049788
ATG16L2	chr11	72814405	72829635
C3	chr19	6677834	6720682
COL5A2	chr2	1.89E+08	1.89E+08
GBP2	chr1	89106132	89126159
GATSL3	chr22	30285117	30289627
ADAR	chr1	1.55E+08	1.55E+08
SPARC	chr5	1.52E+08	1.52E+08
FOSB	chr19	45467994	45475179
SCNN1B	chr16	23302269	23381299
PTPRO	chr12	15322256	15598331
STAG3L5P-PVRIG2P-PILRB	chr7	1E+08	1E+08
TLR3	chr4	1.86E+08	1.86E+08
CLU	chr8	27596916	27614811
TXNIP	chr1	1.46E+08	1.46E+08
TAPBPL	chr12	6452010	6470899

ADM	chr11	10304979	10307402
EIF2AK2	chr2	37105140	37157047
COL12A1	chr6	75084325	75205907
METRNL	chr17_KI270860v1_alt	8451	24134
CLCN6	chr1	11762192	11847783
TDRD7	chr9	97412019	97496123
ACSS2	chr20	34874962	34927966
SLC22A18	chr11_KI270831v1_alt	119555	157362
ANKRD2	chr10	97572440	97583886
FAM20C	chr7_KI270899v1_alt	185289	188053
ERAP2	chr5	96875939	96919702
SLC25A25-AS1	chr9	1.28E+08	1.28E+08
KCNQ3	chr8	1.32E+08	1.32E+08
TACC2	chr10	1.22E+08	1.22E+08
RHOV	chr15	40872213	40874289
BBC3	chr19	47220821	47232766
HLA-C	chr6_GL000256v2_alt	2570706	2574084
RHPN1	chr8	1.43E+08	1.43E+08

OLMALINC	chr10	1E+08	1E+08
NMI	chr2	1.51E+08	1.51E+08
SIPA1L2	chr1	2.32E+08	2.33E+08
FTSJ2	chr7	2234290	2242198
NPIPB4	chr16	21834562	21857756
SLFN5	chr17	35243066	35267742
SECTM1	chr17	82321023	82334045
CPNE7	chr16	89575767	89597246
MMP17	chr12	1.32E+08	1.32E+08
HMGN5	chrX	81113700	81298547
MDK	chr11	46380783	46383837
P3H3	chr12	6828372	6839854
GABBR1	chr6_GL000255v2_alt	867739	898698
KIAA1211L	chr2	98793845	98936221
LOC100419583	chr15_KI270849v1_alt	52356	56232
FRAS1	chr4	78057569	78544269
CTSS	chr1	1.51E+08	1.51E+08
CFI	chr4	1.1E+08	1.1E+08

SRGN	chr10	69088071	69104811
ALPK1	chr4	1.12E+08	1.12E+08
IFITM3	chr11	319672	320914
MAP3K8	chr10	30434020	30461833
NFKBIZ	chr3	1.02E+08	1.02E+08
EHF	chr11	34621040	34663287
SERPINA5	chr14	94581368	94593120
RRBP1	chr20	17613677	17682283
CNTNAP1	chr17	42682613	42699993
ABCG1	chr21	42199688	42297244
RRP36	chr6	43021646	43029599
CXCL3	chr4	74036590	74038773
NOXA1	chr9	1.37E+08	1.37E+08
CELSR3	chr3	48636462	48662915
HAPLN3	chr15	88877284	88895626
LPIN1	chr2	11677578	11827407
PI4KAP1	chr22	18533863	18548828
GUSBP11	chr22	2363	237

		8487	17423
FADS2	chr11	61799624	61867354
BTC	chr4	74746237	74794672
ACSL1	chr4	1.85E+08	1.85E+08
LOX	chr5	1.22E+08	1.22E+08
LMTK3	chr19	48485270	48513189
MUC1	chr1	1.55E+08	1.55E+08
CTAGE15	chr7	1.44E+08	1.44E+08
ZNFX1	chr20	49245900	49289258
ZRSR2	chrX	15790450	15823259
ZBTB22	chr6_GL000255v2_alt	4508831	4512370
COL16A1	chr1	31652246	31704167
ITGB2	chr21	44885953	44929680
VGF	chr7	1.01E+08	1.01E+08
CDH4	chr20_KI270869v1_alt	8602	54269
TNFSF13	chr17	7549057	7561608
IFI16	chr1	1.59E+08	1.59E+08
INSIG1	chr7	1.55E+08	1.55E+0

			8
APOL2	chr22	3622 6208	362 399 54
TRIM56	chr7	1.01E +08	1.01 E+0 8
BTN3A3	chr6	2644 0471	264 534 15
CPT1B	chr22	5056 8860	505 829 99
RRP8	chr11	6599 913	660 365 0
MILR1	chr17	6444 9114	644 686 43
NR4A1	chr12	5202 2831	520 595 07
SP8	chr7	2078 2273	207 868 89
CDK18	chr1	2.06E +08	2.06 E+0 8
LOC729737	chr1	1347 72	140 566
PRSS23	chr11	8679 1058	869 553 98
PHF11	chr13	4949 5664	495 289 81
OBSCN	chr1	2.28E +08	2.28 E+0 8
TSSK3	chr1	3236 2260	323 643 23
RNF213	chr17	8026 0860	804 380 84
HLA-A	chr6_GL0002 56v2_alt	1240 283	124 371 4
ACSF2	chr17	5042 6157	504 748 45

INHBA	chr7	4168 9002	417 793 78
FOXO1	chr13	4055 5663	406 665 97
CEP250	chr20	3545 5401	355 119 74
CSF3	chr17	4001 5360	400 178 13
FYB	chr5	3910 5251	392 706 57
TRIM14	chr9	9808 4352	981 193 53
CYP1B1	chr2	3806 7602	380 761 81
NPAS2	chr2	1.01E +08	1.01 E+0 8
ENKD1	chr16	6766 2946	676 667 25
TRIM21	chr11	4384 896	439 369 6
PPL	chr16	4882 506	493 713 5
RRP7A	chr22	4250 0578	425 198 23
SLC9A9	chr3	1.43E +08	1.44 E+0 8
MAFB	chr20	4068 5847	406 892 40
CIART	chr1	1.5E+ 08	1.5 E+0 8
GOLT1A	chr1	2.04E +08	2.04 E+0 8
PLEKHA7	chr11	1678 7659	170 144 16

ZC3H12A	chr1	3747 4517	374 843 77
ETNK2	chr1	2.04E +08	2.04 E+0 8
BTN3A1	chr6	2640 2236	264 152 16
RAB26	chr16	2148 143	215 416 1
LOC155060	chr7	1.49E +08	1.49 E+0 8
RRNAD1	chr1	1.57E +08	1.57 E+0 8
LAP3	chr4	1757 7303	176 079 67
RRP1	chr21	4378 9536	438 041 02
DENND3	chr8	1.41E +08	1.41 E+0 8
SOCS3	chr17	7835 6776	783 600 79
NEAT1	chr11	6542 2797	654 455 38
DTX3L	chr3	1.23E +08	1.23 E+0 8
MIR4697H G	chr11	1.34E +08	1.34 E+0 8
MCAM	chr11	1.19E +08	1.19 E+0 8
SPACA6P	chr19	5168 5362	517 051 90
KCCAT211	chr3	1.78E +08	1.78 E+0 8
CST6	chr11	6601 1990	660 135 05

JPH2	chr20	44111696	44187578
SMAD6	chr15	66702335	66781999
RRP9	chr3	51933425	51941941
MIR210HG	chr11_KI270832v1_alt	95311	98112
RRP1B	chr21	43659550	43696079
TCN2	chr22	30607082	30627060
LGALS3BP	chr17	78971252	78979979
RRP12	chr10	97356700	97401370
NEDD9	chr6	11183297	11382348
SAMD9	chr7	93099512	93118023
APOL6	chr22	35648376	35668409
PTN	chr7	1.37E+08	1.37E+08
RRP15	chr1	2.18E+08	2.18E+08
SCD	chr10	1E+08	1E+08
FLVCR2	chr14	75574903	75648169
LCN2	chr9	1.28E+08	1.28E+08
CCL20	chr2	2.28E+08	2.28E+08
TAP1	chr6_GL000256v2_alt	4239968	425322

			3
TMPRSS3	chr21	4237 1886	423 968 46
IZUMO4	chr19	2096 868	209 958 4
ATF3	chr1	2.13E +08	2.13 E+0 8
CEACAM1	chr19	4239 7127	426 523 55
HDAC9	chr7	1808 6948	189 973 69
HS3ST2	chr16	2281 4538	229 163 38
MYPN	chr10	6810 6116	682 120 16
CEACAM1 9	chr19	4467 1451	446 843 55
CROCC	chr1	1692 1949	169 729 79
MSH5	chr6_GL0002 56v2_alt	3039 244	306 488 0
ZNF114	chr19	4827 0098	482 876 08
GBP1	chr1	8905 2303	890 653 60
MME	chr3	1.55E +08	1.55 E+0 8
COL8A1	chr3	9963 8595	997 967 33
PARP14	chr3	1.23E +08	1.23 E+0 8
ZBTB12	chr6_GL0002 55v2_alt	3153 756	320 164 9
SP110	chr2	2.3E+ 08	2.3 E+0

			8
CD7	chr17	8231 4869	823 176 04
GMPR	chr6	1623 8579	162 955 49
THEMIS2	chr1	2787 2542	278 866 85
SEMA3B	chr3	5026 7557	502 771 71
HCP5	chr6_GL0002 56v2_alt	2702 060	281 041 0
SYT12	chr11	6702 2718	670 508 63
SLC44A2	chr19	1060 2444	106 445 59
STARD5	chr15	8119 6877	813 241 83
BST2	chr19	1740 2938	174 056 48
PLSCR1	chr3	1.47E +08	1.47 E+0 8
PLEKHA4	chr19	4883 7096	488 686 27
C3AR1	chr12	8058 322	806 635 9
CDKN1C	chr11_KI2708 31v1_alt	1153 96	117 975
SAMSN1	chr21	1448 5227	146 431 07
LAPTM5	chr1	3073 2467	307 578 36
RRP7BP	chr22	4255 3861	425 820 11
ATOH8	chr2	8575 3785	857 913 83

UCA1	chr19	15828946	15836321
NLRC5	chr16	57017073	57083524
RARRES3	chr11	63536800	63546458
SLC2A3	chr12	7919227	7936296
HLA-B	chr6_GL000256v2_alt	2656109	2659450
ODF3B	chr22	50530408	50532579
C19orf66	chr19	10086118	10102749
HLA-F	chr6_GL000256v2_alt	1029598	1055278
CYP1A1	chr15	74719541	74725536
STAT2	chr12	56341597	56360253
PDGFRL	chr8	17576432	17643133
TNFSF14	chr19	6663136	6670588
STAT1	chr2	1.91E+08	1.91E+08
PSMB9	chr6_GL000256v2_alt	4253412	4259100
GCA	chr2	1.62E+08	1.62E+08
TNFAIP6	chr2	1.51E+08	1.51E+08
C5orf56	chr5	1.32E+08	1.32E+08

CYP2J2	chr1	5989<br>3307	599<br>267<br>51
PARP10	chr8	1.44E<br>+08	1.44<br>E+0<br>8
TRIM22	chr11	5689<br>586	571<br>086<br>3
SAMHD1	chr20	3687<br>6120	369<br>518<br>43
IRF9	chr14	2416<br>1212	241<br>665<br>66
DDX60L	chr4	1.68E<br>+08	1.68<br>E+0<br>8
PARP9	chr3	1.23E<br>+08	1.23<br>E+0<br>8
GGT5	chr22	2421<br>9653	242<br>451<br>42
CFB	chr6_GL0002<br>56v2_alt	3247<br>132	325<br>327<br>3
DDX60	chr4	1.68E<br>+08	1.68<br>E+0<br>8
FAM65B	chr6	2480<br>4280	250<br>421<br>68
UBE2L6	chr11	5755<br>1654	575<br>683<br>30
EDN2	chr1	4147<br>8774	414<br>846<br>83
TNFSF10	chr3	1.73E<br>+08	1.73<br>E+0<br>8
TRANK1	chr3	3682<br>6816	369<br>450<br>57
IFI35	chr17	4300<br>6724	430<br>144<br>59
ISG20	chr15	8863<br>5636	886<br>563<br>44

GALNT15	chr3	16174676	16229746
IL24	chr1	2.07E+08	2.07E+08
USP18	chr22	18149990	18177395
IFIH1	chr2	1.62E+08	1.62E+08
OAS3	chr12	1.13E+08	1.13E+08
HERC5	chr4	88457116	88506168
ETV7	chr6	36354220	36387800
C1S	chr12	7060675	7071031
HELZ2	chr20	63558085	63574239
HERC6	chr4	88378738	88443097
CA9	chr9	35673917	35681157
DDX58	chr9	32455301	32526324
CXCL11	chr4	76011179	76112802
APOL3	chr22	36140322	36166177
UBA7	chr3	49805204	49813958
APOL1	chr22	36253070	36267531
IFI44L	chr1	78620381	78646145

SAMD9L	chr7	9313 0053	931 483 88
CXCL10	chr4	7601 1179	761 128 02
OAS1	chr12	1.13E +08	1.13 E+0 8
C1R	chr12	7080 210	709 244 7
IRF7	chr11_KI2708 32v1_alt	1424 47	145 898
RTP4	chr3	1.87E +08	1.87 E+0 8
FGF16	chrX	7744 7674	774 565 22
CMPK2	chr2	6840 552	686 663 5
IFI44	chr1	7864 9791	786 640 78
IFIT3	chr10	8932 7818	893 409 68
DHX58	chr17	4210 1405	421 127 33
HSH2D	chr19	1613 4027	161 585 73
IFIT2	chr10	8930 1948	893 092 76
OASL	chr12	1.21E +08	1.21 E+0 8
ISG15	chr1	1013 466	101 453 9
LAMP3	chr3	1.83E +08	1.83 E+0 8
LGALS9	chr17	2763 1147	276 495 60
IFITM1	chr11	3139	315

		90	272
IFIT1	chr10	8939 2545	894 064 87
IFI6	chr1	2766 6060	276 722 13
RSAD2	chr2	6877 664	689 823 2
OAS2	chr12	1.13E +08	1.13 E+0 8
IFI27	chr14_KI2708 47v1_alt	12252 94	123 126 1

Supplementary Table 4

The sequence, respective amplicon size and Tm of the oligonucleotide primers used in Q-PCR:

Gene Name	Forward primer (5’- 3’)	Reverse primer (5’- 3’)	Tm(ºC)
28SrRNA	TCATCAGACCCCAGAAAAGG	GATTCGGCAGGTGAGTTGTT	58
CDH2	CCATCAAGCCTGTGGGAATC	GCAGATCGGACCGGATACTG	56
ZEB1	AAGAAAGTGTTACAGATGCAGCTG	CCCTGGTAACACTGTCTGGTC	59
CDH1	GTCACTGACACCAACGATAATCCT	TTTCAGTGTGGTGATTACGACGTTA	59
DSM	GCAGCAAAGGGCGGAGAT	TGTTAATGTGCTGCTCCACTGA	59
VIM	ACACCCTGCAATCTTTCAGACA	GATTCCACTTTGCGTTCAAGGT	59
18SrRNA	GATTCCGTGGGTGGTGGTGC	AAGAAGTTGGGGGACGCCGA	60
ITGB6	GGACAGCAAGAATGAATAC	CTTGTTCTTGGGTTACAG	55
ITGB2	GTGAAGACCTACGAGAAA	CCTGGAGGAGAGTTTATT	55
ITGA6	TAGAGATGGAGAAGTTGG	GCAATGCCAAACATAGA	55
MCAM	CTGTCCTCTATTTCCTCTA	GGAGCTTATCTGACTTAAC	55
KRT14	CACCAAGTATGAGACAGAG	CTCTCAATCTGCATCTCC	57
LAMA5	CCTGGATAAATCCTATGAC	TGTTATAGAAGAGGGAGAG	55
LAMC2	TCTCTGAAGAGTGAGATG	CTCTGGTATCAACCTTCT	55
COL12A1	CACTGGAGAACTTGATTC	CTCTTGGATTCCCTCTAA	55
COL4A2	GTGTGGAGGAAGAGATTG	CCGGGAATCCTTGTAATC	57
COL8A1	CAGTATGGCAAAGAGTATC	CTCGTAAACTGGCTAATG	55

GLUT3	CTTTAGAGTGTCCAGCTA	CCTTGAAGATTCCTGTTG	55
GLUT1	CTGAGCATCATCTTCATC	GCTTCTTTAGCACACTC	55
HK1	GCTTCACGTTCTCATTT	CTTAGTAAGGTGACTACATC	55
HK2	TGAGATTGTCCGTAACA	CTCAATCTGAGACAAGAAC	55
LDHA	GATGATGTCTTCCTTAGTGTTC	AGTGTATCTGCACTCTTCTT	59
LDHB	TAAAGGAGAAATGATGGATCTG	GCAGTTACCACTACAATCTTA	58
ENO1	GCTGAAGACTGCTATTG	GAAGTCCAGGTCATACT	58
GAPDH	TGCACCACCAACTGCTTAGC	GGCATGGACTGTGGTCATGAG	58
PFKFB4	GCTCAGGATAGAGACAGAAA	TCTTGGAGATGTAGGTCTTG	58
PFKFB3	AATGAGGAAGCCATGAAAG	TCTAGTAGTATTGGTGGCAT	58
PFKP	TCATTTACCAGCTGTATTC	GGTTCCAAAGTTTCTATCA	55
PFKL	CATGGGCATTTATGTGG	CTGGATGATGTTGGAGA	55
PFKM	TTCATGTGTGTGATTCAG	CTTCAAGATGTCTGTCTTT	55
SNAIL	TCGGAAGCCTAACTACAGCGA	AGATGAGCATTGGGCAGCGAG	60
MMP2	TGGTGAGATCTTCTTCTTCA	CGTATACCGCATCAATCTTT	58

Supplementary Table 5

List of antibodies used in Western blot and immunofluorescence staining:

Name of antibody used	Application	Dilution used	Company	Catalogue No.
Laminin	IF	1:400	Novus	NB300-144
Ki67	IF<br>IF	1:500<br>1:500	CST<br>Novus	3396S<br>NB110-89719
GAPDH	Western blot	1:2000	Bio Bharati	BB-AB0060
VIM	Western blot<br>IF	1:1000<br>1:400<br>1:100	Abcam<br>Cell Signalling Technology (CST)	ab92547<br>D21H3
E-cadherin	Western blot<br>IF	1:1000<br>1:100<br>1:400	Abcam<br>Cell Signalling Technology	ab40772<br>24E10
α-Tubulin	Western blot	1:4000	Bio Bharati	BB-AB0118
Rabbit-HRP	Western blot	1:6000	Cell Signalling Technology	7074S
Alexa Fluor-488	IF	1:500	Molecular Probes	A11034
Phalloidin-iFluor 488	IF	1:1500	Abcam	ab176753
Integrin A6	IF	1:1000	Abcam	ab20142
PFKFB4	Western Blot	1:1000	ABclonal	A17915
PFKP	Western Blot	1:1000	ABclonal	A12160
FGF16	IF	1:50	Thermo Scientific	PA5-50664

Supplementary Table 6

List of clinico-pathological information of human breast tumor tissue used for QPCR and IHC.

SAMPLE	AGE AT DIAGNOSIS	MOLECULAR SUBTYPE	TREATMENT STATUS
BC 96	57	ER-PR-HER2+	NAÏVE
BC128	55	ER-PR-HER2+	NAÏVE
BC81	46	ER+PR+HER2-	NAÏVE
BC85	38	ER+PR+HER2+	NAÏVE
BC84	34	ER+PR+HER2-	NAÏVE
BC123	35	ER+PR+HER2equi	NAÏVE
BC88	47	ER+PR+HER2-	NAÏVE
BC102	31	ER+PR+HER2-	NAÏVE
BC35	40	ER-PR-HER2-	NAÏVE
BC57	55	ER-PR-HER2equi	NAÏVE
BC16	46	ER-PR+HER2-	NAÏVE
BC75	45	ER+PR+HER2+	NAÏVE
BC118	24	ER+PR+HER2-	NAÏVE
BC40	40	ER+PR-HER2+	NAÏVE
BC29	48	ER+PR+HER2-	NACT
BC129	26	ER-PR-HER2-	NAÏVE
BC79	31	ER-PR-HER2+	NAÏVE
BC94	56	ER-PR-HER2+	NAÏVE
BC32	35	ER+PR+HER2equi	NACT
BC28	48	ER-PR-HER2+	NAÏVE
BC191	61	ER+PR+HER2-	NAÏVE

BC193	65	ER+PR+HER2equi	NAÏVE
BC176	51	ER+PR+HER2equi	NAÏVE
BC172	55	ER-PR-HER2+	NAÏVE
BC220	39	ER+PR+HER2-	NAÏVE
BC226	58	-	NAÏVE

Figure 1G

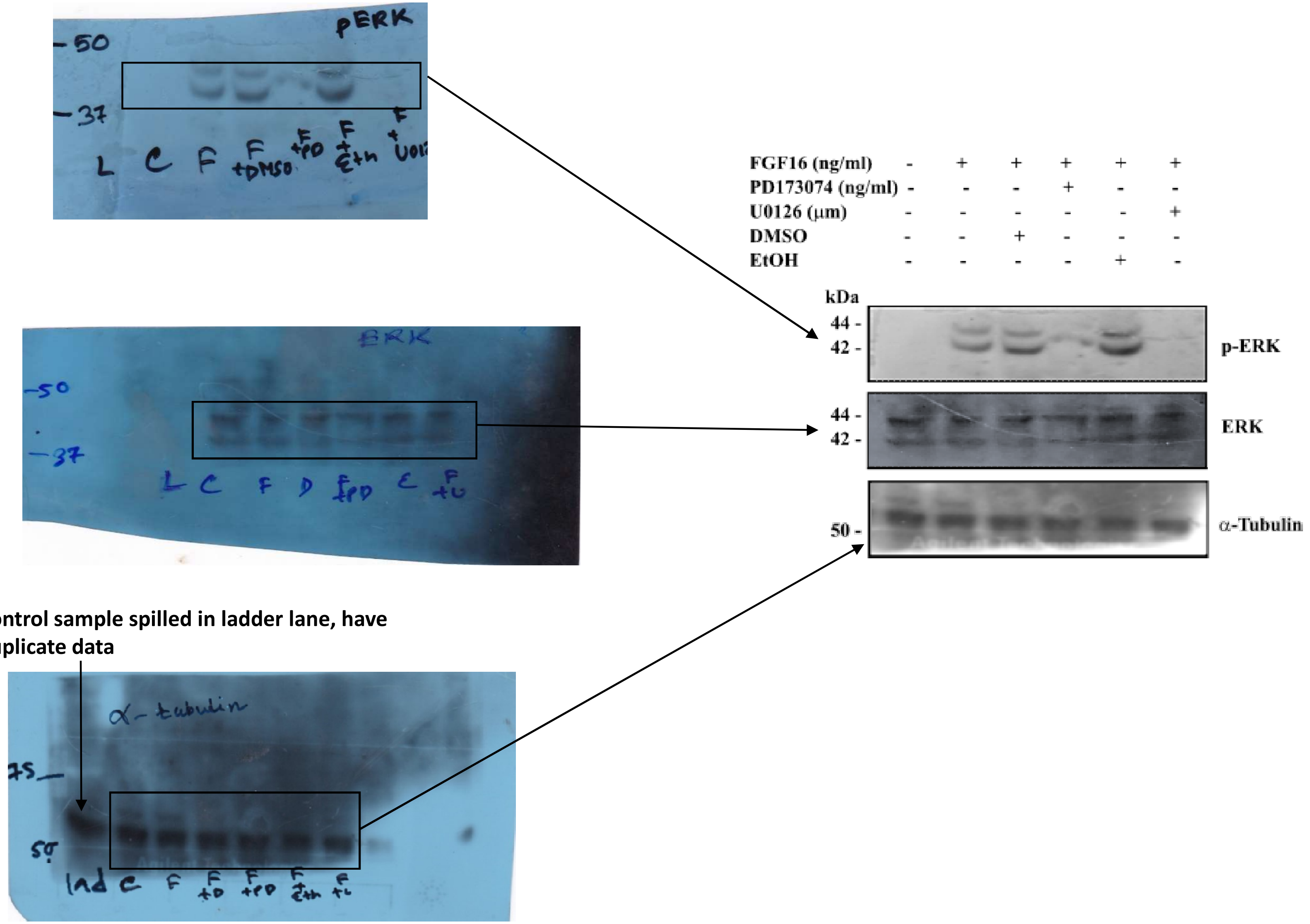
pERK
50
37
ERK
α-tubulin
Control sample spilled in ladder lane, have duplicate data
FGF16 (ng/ml) - + + + + +
PD173074 (ng/ml) - - - + - -
U0126 (μm) - - - - - +
DMSO - - + - - -
EtOH - - - - + -
kDa
44 -
42 -
p-ERK
44 -
42 -
ERK
50 -
α-Tubulin

Figure 1D

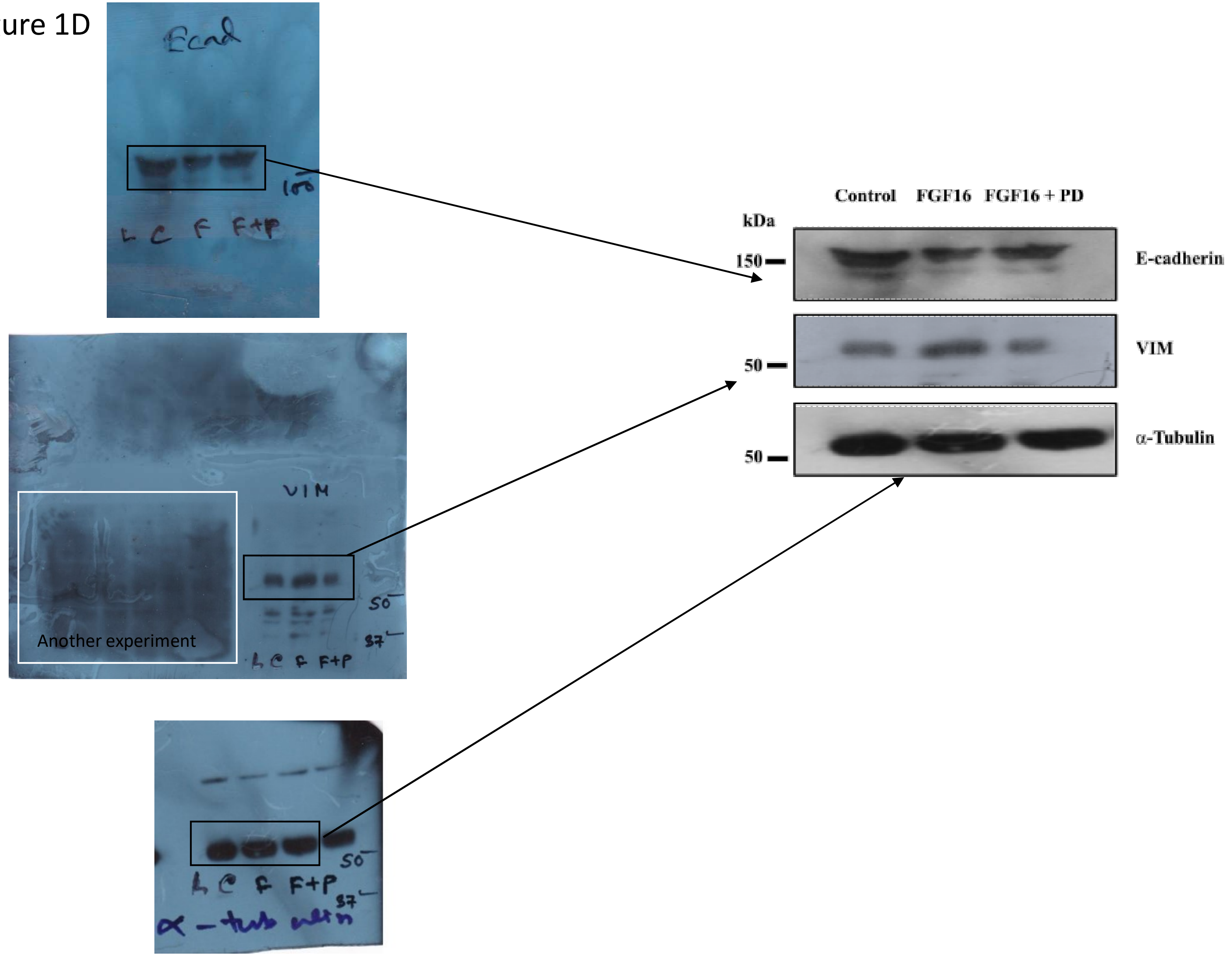
Control FGF16 FGF16 + PD
kDa
150
50
50
E-cadherin
VIM
α-Tubulin
Another experiment

Supp Figure 1E

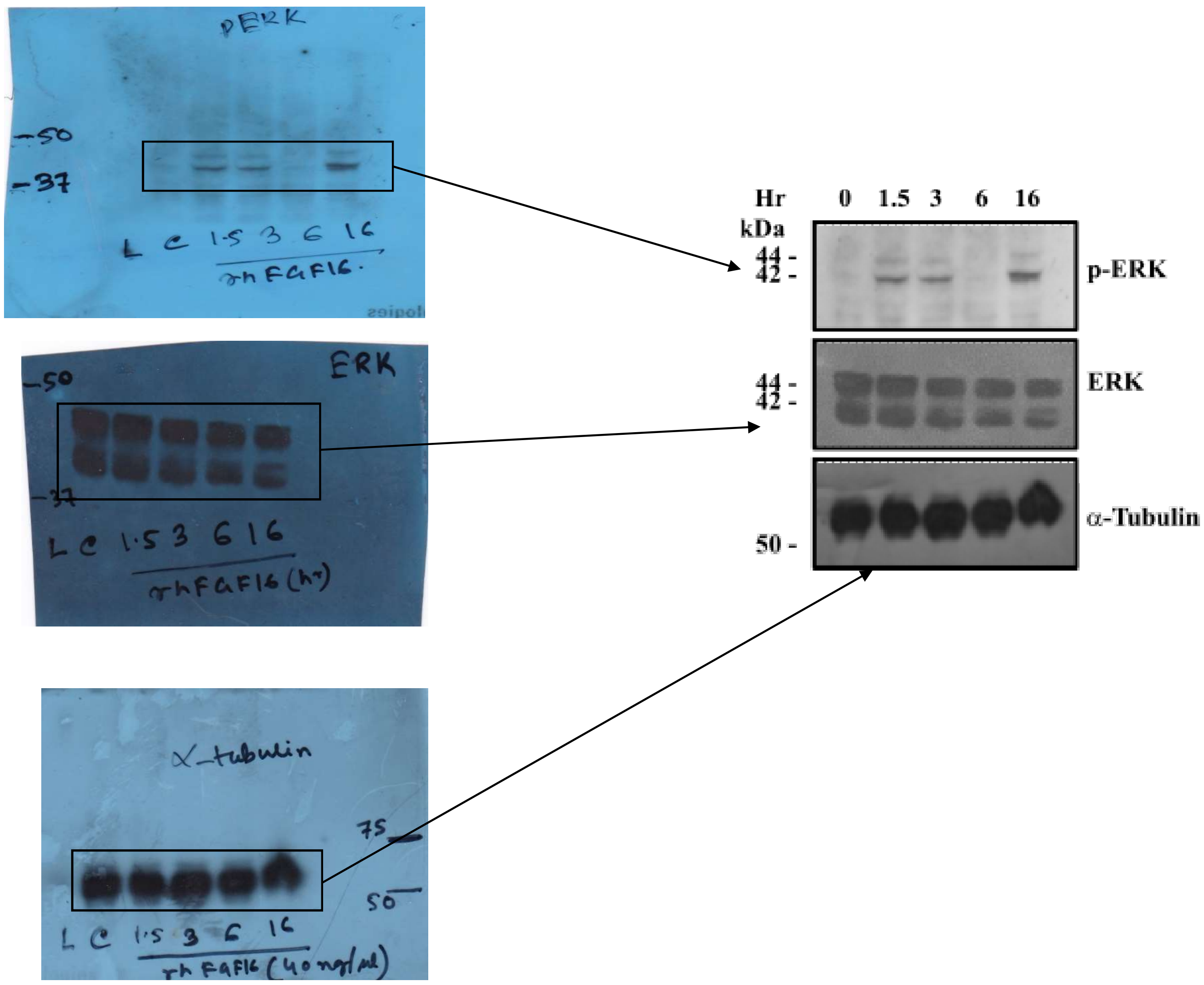

Supp Figure 1F

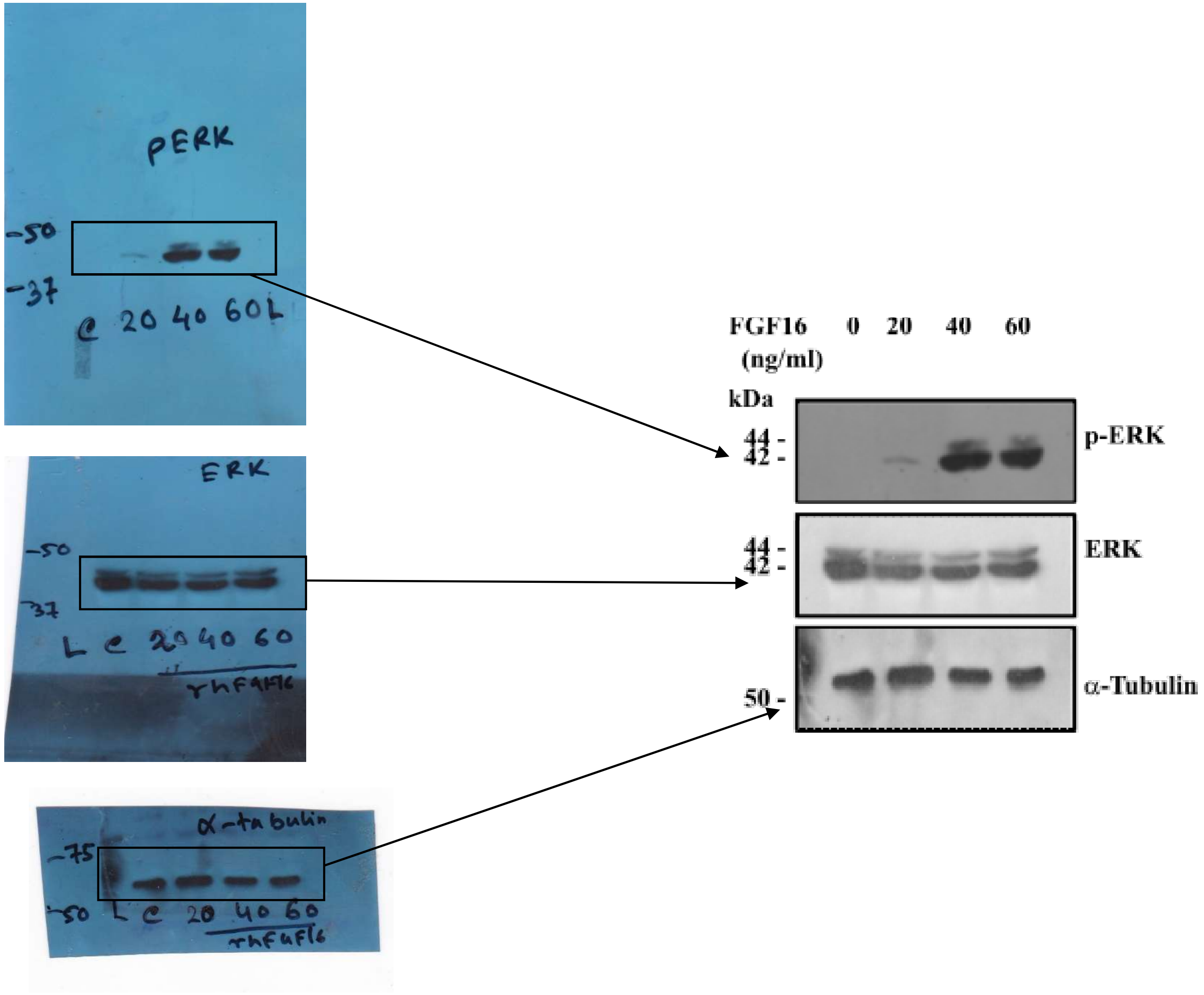

Supp Figure 1G

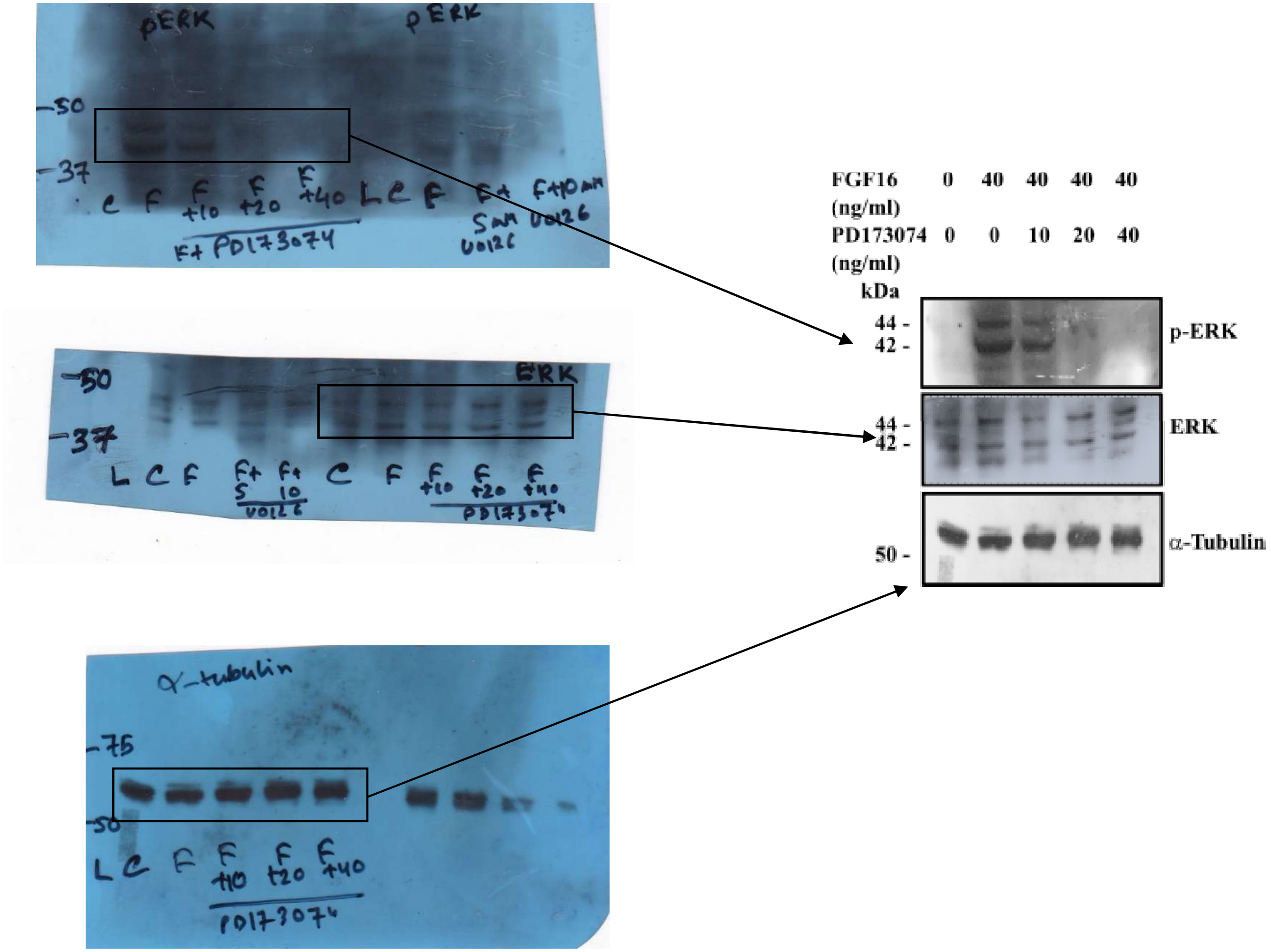

Supp Figure 1H

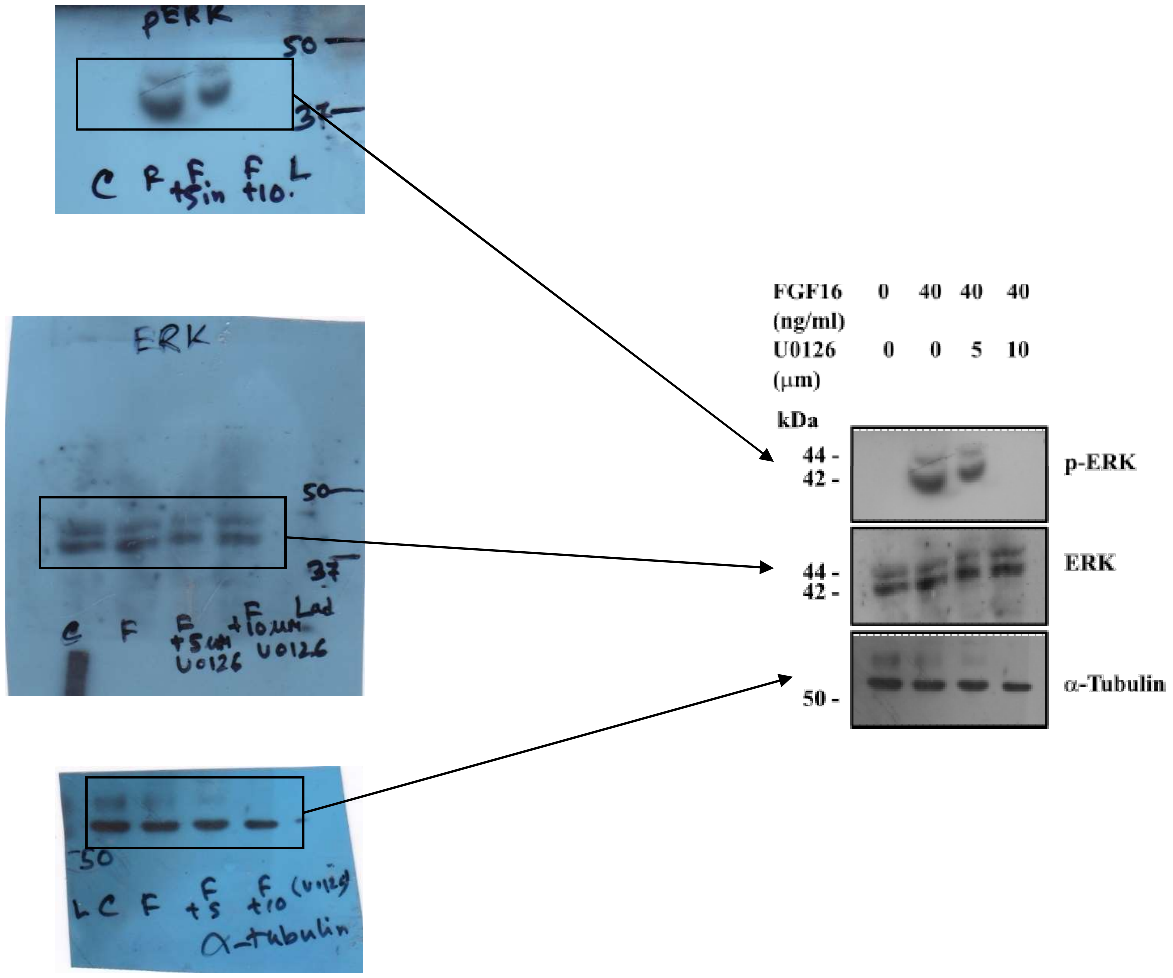

Supp Figure 5B

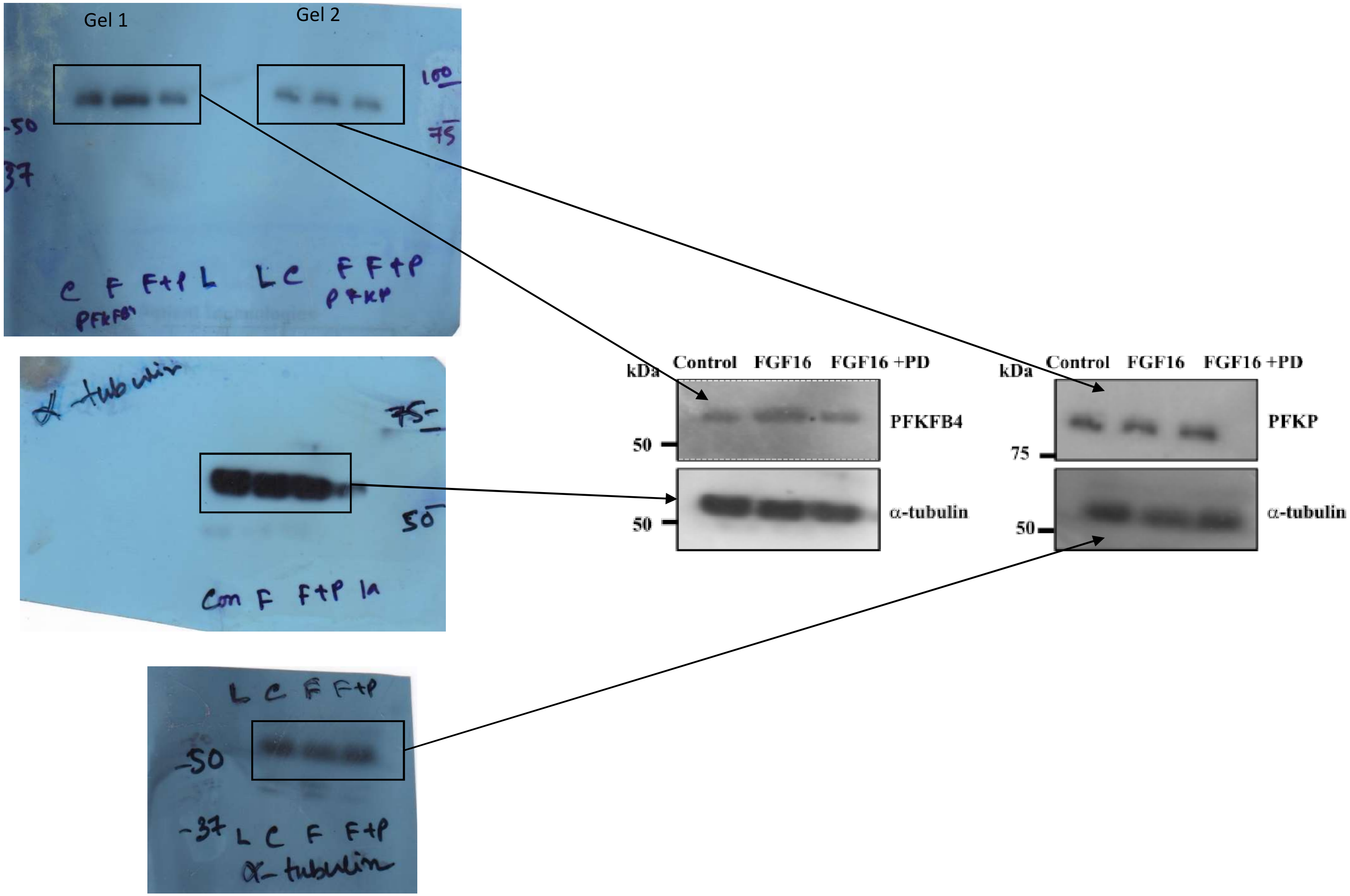

Supp Figure 5D

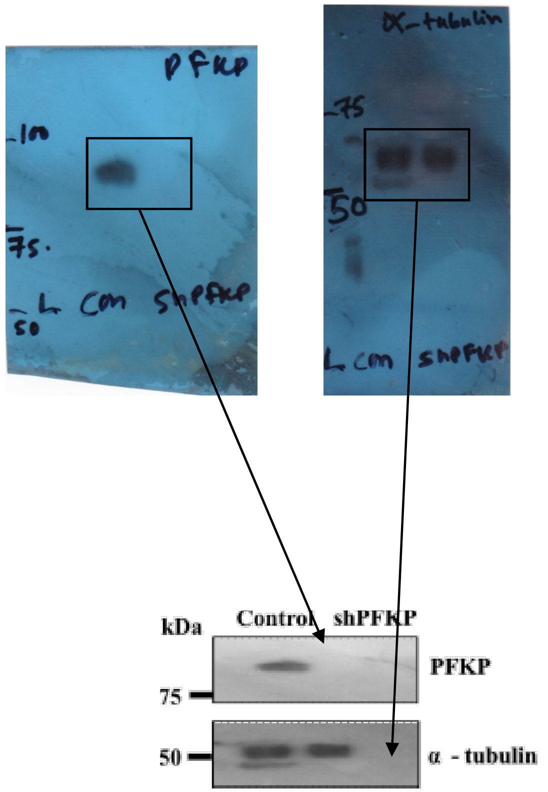

Supp Figure 5E

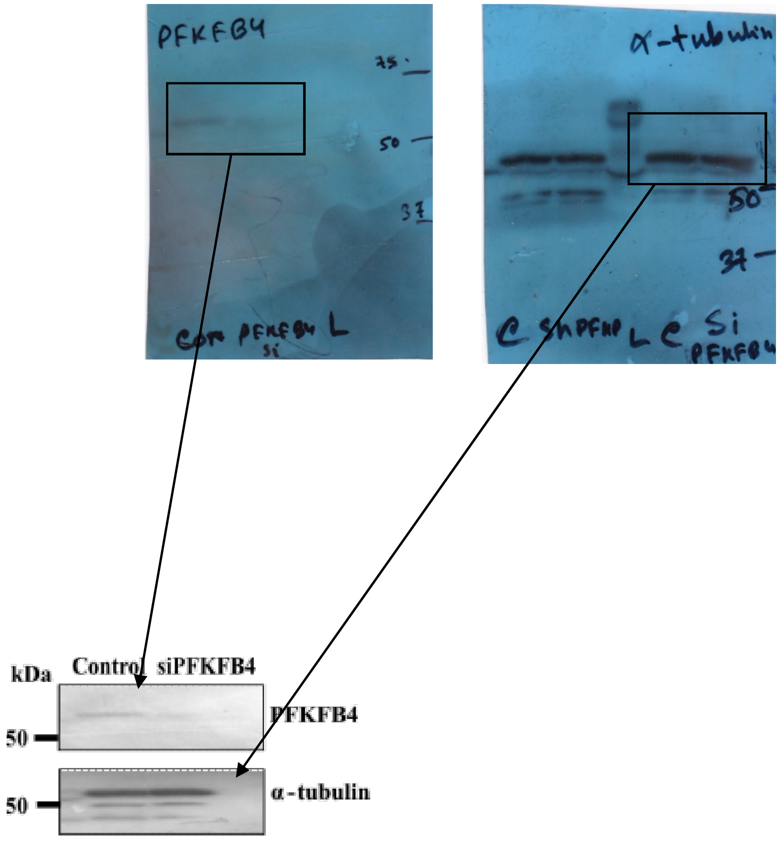

Supplement: Supplementary Figures S1-S5 and Tables S1-S6 [file BSR-2023-0677_supp.pdf]
